# Expansion microscopy allows high resolution single cell analysis of epigenetic readers

**DOI:** 10.1093/nar/gkac521

**Published:** 2022-06-18

**Authors:** Aline Acke, Siska Van Belle, Boris Louis, Raffaele Vitale, Susana Rocha, Thierry Voet, Zeger Debyser, Johan Hofkens

**Affiliations:** Laboratory for Photochemistry and Spectroscopy, Department of Chemistry, KU Leuven, Leuven, Flanders, Belgium; Laboratory for Molecular Virology and Gene Therapy, Department of Pharmaceutical and Pharmacological Sciences, KU Leuven, Leuven, Flanders, Belgium; Laboratory for Photochemistry and Spectroscopy, Department of Chemistry, KU Leuven, Leuven, Flanders, Belgium; Division of Chemical Physics and NanoLund, Lund University, Lund, Sweden; Dynamics, Nanoscopy and Chemometrics (DYNACHEM) Group, U. Lille, CNRS, LASIRE, Laboratoire Avancé de Spectroscopie pour les Interactions, la Réactivité et l’Environnement, Cité Scientifique, F-59000Lille, France; Laboratory for Photochemistry and Spectroscopy, Department of Chemistry, KU Leuven, Leuven, Flanders, Belgium; Department of Human Genetics, KU Leuven, Leuven, Flanders, Belgium; LISCO, KU Leuven Institute for Single-Cell Omics, Leuven 3000, Belgium; Laboratory for Molecular Virology and Gene Therapy, Department of Pharmaceutical and Pharmacological Sciences, KU Leuven, Leuven, Flanders, Belgium; Laboratory for Photochemistry and Spectroscopy, Department of Chemistry, KU Leuven, Leuven, Flanders, Belgium; Max Plank Institute for Polymer Research, Ackermannweg 10, Mainz, D-55128, Germany; LISCO, KU Leuven Institute for Single-Cell Omics, Leuven 3000, Belgium

## Abstract

Interactions between epigenetic readers and histone modifications play a pivotal role in gene expression regulation and aberrations can enact etiopathogenic roles in both developmental and acquired disorders like cancer. Typically, epigenetic interactions are studied by mass spectrometry or chromatin immunoprecipitation sequencing. However, in these methods, spatial information is completely lost. Here, we devise an expansion microscopy based method, termed Expansion Microscopy for Epigenetics or ExEpi, to preserve spatial information and improve resolution. We calculated relative co-localization ratios for two epigenetic readers, lens epithelium derived growth factor (LEDGF) and bromodomain containing protein 4 (BRD4), with marks for heterochromatin (H3K9me3 and H3K27me3) and euchromatin (H3K36me2, H3K36me3 and H3K9/14ac). ExEpi confirmed their preferred epigenetic interactions, showing co-localization for LEDGF with H3K36me3/me2 and for BRD4 with H3K9/14ac. Moreover addition of JQ1, a known BET-inhibitor, abolished BRD4 interaction with H3K9/14ac with an IC_50_ of 137 nM, indicating ExEpi could serve as a platform for epigenetic drug discovery. Since ExEpi retains spatial information, the nuclear localization of marks and readers was determined, which is one of the main advantages of ExEpi. The heterochromatin mark, H3K9me3, is located in the nuclear rim whereas LEDGF co-localization with H3K36me3 and BRD4 co-localization with H3K9/14ac occur further inside the nucleus.

## Graphical Abstract

**Graphical Abstract:**
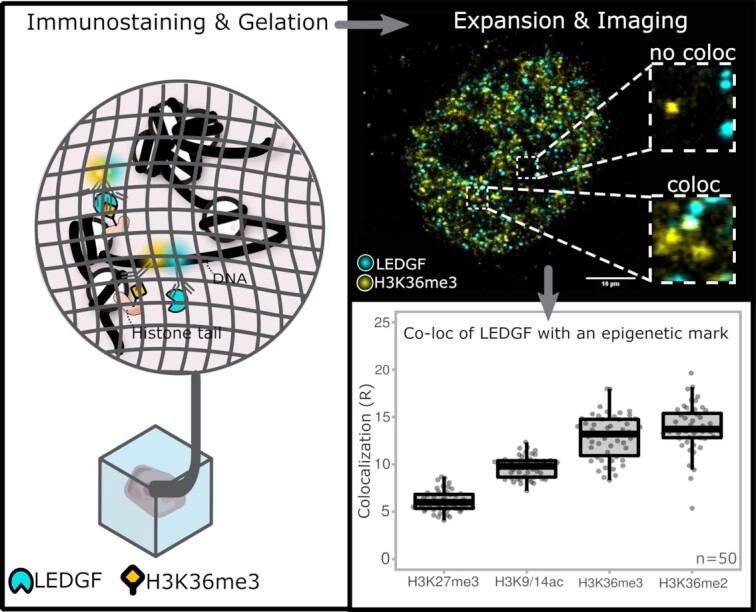
Co-localization of an immunostained epigenetic reader (LEDGF) and histone modification (H3K36me3) using expansion microscopy to image and quantify interactions of chromatin binding proteins at different post-translational modifications, with retained spatial information in a single cell or in short, expansion microscopy for epigenetics (ExEpi).

## INTRODUCTION

Epigenetics is defined as the study of heritable changes in phenotype that are not related to alterations in the DNA sequence *per se*. As such, epigenetics refers to different levels of modifications involving methylation of the DNA sequence in CpG dinucleotide contexts, post-translational modifications (methylation, acetylation, etc.) of histones, as well as non-coding (nc)RNA-mediated chromatin alterations, all leading to a dynamic yet tight regulation of gene expression (1). Histone modifications, DNA methylation and chromatin remodelling define the epigenome, which serves as a conceptual framework to understand transcriptional regulation in normal development and human disease (2). Histone proteins can be modified on their core or both N-terminal and C-terminal tails by acetylation, methylation, phosphorylation, etc. of amino acids (3). The formation of such post-translational modifications (PTMs) on histones is a dynamic process and is carried out by a set of enzymes referred to as epigenetic writers, readers and erasers that can add, read or remove specific marks (4).

Epigenetic writer enzymes like histone acetyltransferases or histone methyltransferases will add acetyl and methyl groups, respectively, on lysine residues of histones whereas eraser enzymes such as histone deacetylases and demethylases remove these modifications (5). Histone acetylation of lysine residues will neutralize the positive charge of the histone core and as a result make the DNA more accessible for transcription factors facilitating gene transcription (6). Histone methylation on the other hand, is associated with both gene activation and repression depending on the location and number of methyl groups present. For example, tri-methylation of the 27th lysine residue in histone 3 or in short H3K27 can lead to transcriptional suppression (7), whereas H3K36 methylation stimulates gene expression and plays a role in DNA repair and mRNA splicing (8).

Histone modifications function as chromatin recognition marks for specific proteins called epigenetic readers. These proteins can form complexes with a diverse selection of transcription factors and regulate a range of different processes inside cells like DNA replication, gene transcription and chromatin remodeling (9). These processes are pivotal in development, whereby a totipotent fertilized egg divides and differentiates in pluri- to uni-potent cell types cooperating in a multi-cellular organism. As a result, mutations in chromatin remodeling proteins have been linked to neurodevelopmental disorders like the Autism Spectrum Disorder (ASD) (4). Also, alterations in the binding sites of epigenetic reader proteins were shown to drive tumor development in e.g. leukemia (10). Therefore, knowledge about their function and interaction with specific epigenetic modifications does not only help to achieve a better understanding of oncogenesis but also makes it possible to uncover targets for specific cancer treatment (5).

Usually, epigenetic modifications and their interacting proteins are investigated by chromatin immunoprecipitation (ChIP) (11). After fixation of all protein DNA complexes and fragmentation of the DNA, an immunoprecipitation (IP) step will capture specific proteins bound to the DNA by making use of antibodies. The IP is followed by a DNA analysis through sequencing (ChIP-seq) or microarrays (ChIP-chip) (12). Additionally, the development of single-cell ChIP-seq (scChIP-seq) allows to uncover specific histone PTMs and epigenetic reader locations in the genome of individual cells and avoid averaging of the chromatin landscape (13). In a similar fashion, single-cell Cleavage Under Target and Tagmentation (scCUT&Tag) was also recently developed to study histone modifications and binding of transcription factors in the mouse brain (14). Next to these ChIP-based assays, mass spectrometry (MS) is also regularly used to discover PTMs found on histones and their association with proteins (15). Recently, the combination of MS with engineered chromatin readers (eCRs) enabled a detailed investigation of several protein interactions such as BRD4 with histone PTMs like H3K4me3, H3K9me3 and H3K27me3 (16).

In spite of these advances, it is still challenging to unravel the relation between these epigenetic readers and modifications while preserving their spatial organization. Fluorescence microscopy could be the answer to this problem as it allows the study of the chromatin architecture *in situ* through fluorescent staining of DNA, histone proteins and/or histone modifications (17). However, the diffraction limit (∼200–300 nm) of a conventional microscopy system does not allow for a detailed read-out of the events that occur at nanoscale. Although super-resolution (SR) fluorescence microscopy techniques could help to overcome this limit, they remain costly and require specific technical expertise. This can be avoided by making use of expansion microscopy (ExM). By implementing expandable hydrogels, ExM expands the sample of interest up to 4 times its original size in an isotropic fashion and as such, a lateral resolution between ∼70-80 nm is achieved by confocal fluorescence microscopes (18–20).

Here, we aimed at using expansion microscopy to map interactions between histone modifications and specific epigenetic readers in more detail. Even though expansion microscopy does not accomplish single-molecule resolution, the improvement still enables studies at the single-cell level without loss of the aforementioned spatial arrangement. As a test case, we investigated the epigenetic reader lens epithelium derived growth factor (LEDGF) which is a potential drug target as it plays a role in HIV infection, mixed lineage leukemia and other cancers (21). LEDGF consists of two isoforms, p75 and p52, obtained by alternative splicing from a single gene (22) and interaction of both isoforms with methylated H3K36 occurs via the Pro-Trp-Trp-Pro (PWWP) domain (21). In this research, a co-localization analysis is used to demonstrate the known interaction of LEDGF with H3K36me3/2, but also other modifications such as H3K27me3 and H3K9/14 di-acetylation were investigated.

Apart from LEDGF, we also studied bromodomain containing protein 4 (BRD4) which is a member of the bromodomain and extraterminal (BET) protein family and a histone acetylation reader that binds acetylated lysine residues via two bromodomains, BD1 and BD2 (23). BRD4 is involved in the regulation of transcription activation by interaction with transcription factors after binding to acetylated promoter or super enhancer regions, which are strongly enriched for binding of transcriptional coactivators (23). Inhibition of BRD4 has been shown to suppress both prostate and breast cancer cells and is therefore an interesting target to suppress cancer development (24). A commonly used BET inhibitor is the molecule JQ1, which mimics the shape of the acetyl-lysine binding pocket in BRD4 (25). This results in competitive binding, inhibiting BRD4 from interaction with the chromatin and as such reduces tumor growth. In line with previous works (26), BRD4 co-localization with H3K9/14 di-acetylation was corroborated and the concentration dependent inhibition of a BET-inhibitor (JQ1) of the interaction of BRD4 with H3K9/K14 di-acetylation was demonstrated. For both epigenetic readers, co-localization between the proteins and epigenetic modifications was analyzed based on pixel overlap in expanded samples. This method, referred to as Expansion Microscopy for Epigenetics or ExEpi, will enable the study of epigenetic readers while retaining spatial information and possibly serve as a platform for epigenetic drug discovery.

## MATERIALS AND METHODS

Details on used reagents and compounds and their suppliers can be found in the supplementary information (Supplementary Table 11).

### Cell culture

HeLaP4 cells were obtained through the AIDS Research and Reference Reagent Program, Division of AIDS, NIAID, NIH from Dr. Richard Axel (27) and tested negative for mycoplasma contamination. Cells were cultured in high glucose (4.5 g/l), glutamine free, phenol red-free Dulbecco's Modified Eagle Medium (DMEM) supplemented with 10% (v/v) fetal bovine serum, 50 μg/ml gentamicin, 500 μg/ml geneticin and 1% glutamax in 5% CO_2_ at 37°C. Once cells were 70% confluent, they were washed with 1× Dulbecco's phosphate buffered saline (DPBS) followed by detachment with 10× TrypLETM Enzyme and seeded inside T25 flasks at a concentration of 400 × 10³ cells per flask. The same protocol was applied for HeLaP4 LEDGF/p75 depleted (LEDGF KD) and back complemented (LEDGF BC) cells with the addition of 100 μg/ml zeocin for the LEDGF KD cells and both 5 μg/ml blasticidin and 100 μg/ml zeocin for the LEDGF BC cells to the growth medium.

### Western blotting

For the cell lysates, two million cells were washed in PBS, pelleted (5’, 1000 rpm) and lysed using RIPA buffer (ThermoFisher Scientific). The protein concentration was determined using the PierceTM BCA protein assay kit (ThermoFisher Scientific) and 20 μg were loaded onto an 18-well 4–15% Criterion™ TGX™ Precast Midi Protein Gel (Biorad) in 1× Novex Tris-glycine SDS Running Buffer (ThermoFisher Scientific). For the nucleosomes, 0.25 μg of biotinylated H3K36me2 (tebu bio) or biotinylated H3K36me3 (tebu bio) were ran in 1× NuPage MES SDS Running Buffer (ThermoFisher Scientific) to better separate the lower molecular weight proteins. The PageRuler™ Prestained Protein Ladder (ThermoFisher Scientific) was used as size standard. After the separated proteins were transferred to a nitrocellulose membrane by the TransBlot Turbo Transfer System (Biorad), the membrane was incubated for 10 min in ponceau S (Sigma), washed in distilled water and imaged using an ImageQuant 800 (GE Healthcare). The membrane was blocked in 5% (w/v) milk for 1 h and incubated overnight with one of the following primary antibody dilutions: 1/2000 LEDGF/p75 (A300-848A, Bethyl); 1/2000 LEDGF (611715, BD Bioscience); 1/1000 or 1/2000 H3K36me2 (ab176921, Abcam); 1/1000 or 1/2000 H3K36me3 (ab9050, Abcam). After three washes of 5 min in PBS-T (PBS + 0.1% Tritron X), the membrane was incubated with goat HRP-conjugated secondary antibodies against rabbit or mouse (diluted 1/10 000) for one hour at room temperature. After washing, the membrane was analyzed using a Clarity ECL (biorad) and the ImageQuant 800.

### Immunostaining and anchoring

Cells were seeded into 29 mm glass-bottom dishes at a concentration of 300 × 10³ cells per dish and cultured at 37°C in 5% CO_2_ humidified atmosphere in the correct growth medium overnight. Next, cells were incubated with 4% paraformaldehyde (PFA) at room temperature for 10 min followed by three washing steps (each lasting 5 min) with 1× DPBS. After fixation, cells were permeabilized with 0.2% Triton X-100 and 2% bovine serum albumin (BSA) diluted in 1× DPBS for 30 min at room temperature and again washed 3× with 1× DPBS for 5 min. A blocking step before primary antibody incubation was carried out by addition of blocking buffer (10% fetal bovine, 0.2% Tween-20 and 0.2% Triton X-100 in 1× DPBS) for 15 min. Afterwards, the cells were emerged with primary antibodies (concentrations of different primary antibodies are given in Supplementary Table 11) in blocking buffer and incubated overnight at 4°C in dark. Primary antibodies were removed with three washes with 1× DPBS for 5 min followed by a 3-h incubation with secondary antibodies (Goat Anti-Mouse IgG, Alexa488; Goat Anti-Rabbit IgG, Atto647N) at room temperature diluted at 1:500 in blocking buffer with 5% goat serum. Finally, cells were washed twice for 5 min with blocking buffer, supplemented with 1:1000 DAPI (4′,6-diamidino-2-phenylindole) and eventually washed three times with 1× DPBS for 5 min. After the staining, cells were incubated with 0.1 mg/ml Acryloyl-X, SE (6-((acryloyl)amino)hexanoic acid, succinimidyl ester) overnight at room temperature and finally washed twice with 1× DPBS for 15 min before storage at 4°C.

### Gelation, digestion and expansion

Gelation stock solution (1× PBS, 2 M NaCl, 8.625% (w/w) sodium acrylate, 2.5% (w/w) acrylamide and 0.15% (w/w) *N*,*N*’-methylenebisacrylamide) was made and stored in aliquots at –20°C. Before use, an aliquot was thawed on ice and 200 μl per dish were used to briefly pre-incubate cells with the solution. Next, the gelation stock solution was enriched on ice with 0.2% tetramethylenediamine and 0.2% ammonium persulfate and, after removing all previous solution from the cells, 200 μl of the enriched one were added. The samples were transferred to a container and purged with nitrogen gas followed by gelation at 37°C for 1.5 h. After gelation, the gel was cut in an asymmetrical shape with a razor blade and incubated overnight at room temperature in 1 ml of digestion buffer (50 mM Tris, 1 mM EDTA (pH 8), 0.5% Triton X-100, 0.8 M guanidine HCl) with the addition of proteinase K diluted to 8 U/ml. The day after, the samples were transferred to a glass-bottom 6-well plate and expanded in 3 ml of deionized water, refreshing water 4–5 times every hour until the gels no longer expanded.

### Fluorescence imaging

Imaging was performed with a HCPLAPO CS2 63× water immersion objective (NA 1.2) on an inverted Leica true confocal scanner SP8 X system (Wetzlar, Germany). Nuclear stainings with DAPI were imaged using a 405 nm pulsed diode laser. Alexa 488 and Atto 647N were excited at 499 and 647 nm, respectively, by making use of a supercontinuum white light laser (SuperK EXTREME/FIANIUM, NKT photonics, Birkerød, Denmark), and filtered by a notch filter (Leica Microsystems). The correct emission signal was detected by a Leica Hybrid Detector and separated by prism dispersion. A 0.5–12.0 ns gating was applied to minimize reflection when imaging the Atto647N fluorophore. For all samples, both gain and pinhole size (1 airy unit (AU)) were kept constant and all images were obtained taking the Nyquist criterion into account. The laser power for pre-expansion detection was between 3 and 18 μW (DAPI = 4.5 μW; Alexa 488 = 3.3 μW and Atto 647N = 18 μW) while post-expansion an increase in power was needed due to the know fluorophore dilution in expansion (DAPI = 37 μW; Alexa 488 = 19 μW and Atto 647N = 80 μW). *Z*-stacks of around 9 - 11 slices were collected with a distance of 200 nm between each *Z*-slice.

### Image processing and data-analysis

Images were acquired by Leica Application Suite X and pre-processing (such as a 180° rotation of the LEDGF channel for the negative control) was performed by ImageJ (FIJI). Gel drift was corrected by making use of Huygens Professional Object Stabilizer (Scientific Volume Imaging). Intensities of LEDGF staining for wild-type, LEDGF/p75 depleted and back complemented HeLaP4 cells were quantified with a FIJI script (Supplementary Table 12) whereas co-localization, distance calculations and PCC analysis were performed by means of a MATLAB code developed in-house (the source code can be found at https://github.com/BorisLouis/Colocalization). For non-expanded cells the following user input was used: locROI = 10; chi2 = 15; FWHM = 2 whereas for expanded cells the user input was: locROI = 20; chi2 = 70; FWHM = 10. The co-localization analysis was performed on Z-stacks of expanded cells consisting of 9–11 Z-slices with a Z-step size of 0.2 μm. Co-localization ratios based on the mean p-value output were calculated by dividing the number of co-localizing particles by the number of non-co-localizing particles and multiplying this by 100. Finally, all ratios were plotted in a boxplot using the PlotsOfData tool available at https://huygens.science.uva.nl/PlotsOfData/ and statistically significant differences were determined via one-way ANOVA.

## RESULTS

### Concept and validation of expansion microscopy for Epigenetics (ExEpi)

**Figure 1.**
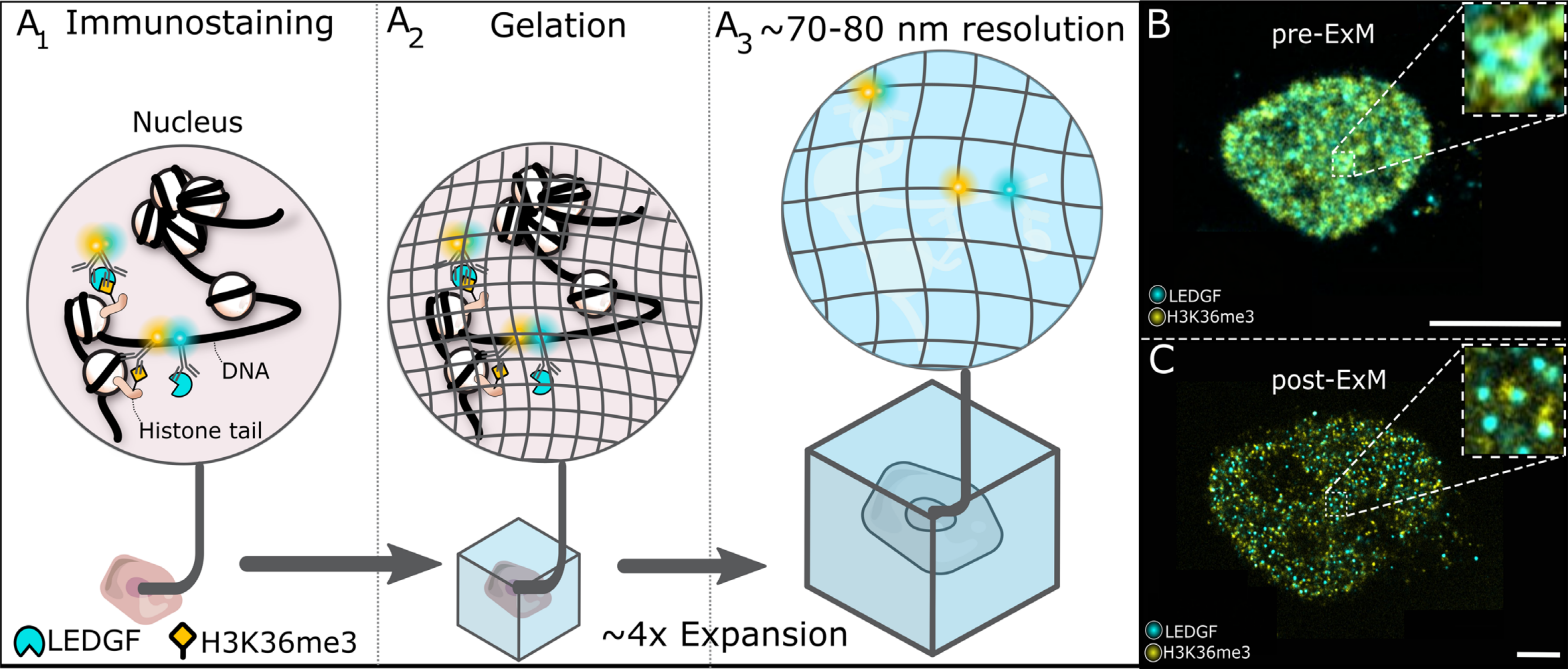
ExEpi: schematic illustration of the concept and typical images obtained pre- and post-expansion. (**A**) Immunolabeling of the chromatin reader LEDGF and the epigenetic modification (H3K36me3) in permeabilized and fixed cells using fluorescently labelled antibodies (A_1_) is followed by a gelation (A_2_) and expansion step (A_3_) to improve resolution showing an actual overlap and thus interaction between LEDGF (blue) and H3K36me3 (yellow) molecules in the top, whereas the two bottom molecules no longer show co-localization in comparison to the sample before expansion. (**B**) Composite immunofluorescence image of the LEDGF protein in cyan and the H3K36me3 modification in yellow before gelation and expansion in the nucleus of a HeLaP4 cell. The right upper corner depicts a zoom of the boxed area. (**C**) Same cell as in (B) but after the gelation and expansion process with improved resolution. The presented images (B-C) are single optical sections. Scale bars: 10 μm (B, C). Details on used antibodies and dilutions can be found in SI Table 11.

To investigate the association of chromatin readers with epigenetic marks and study co-localization while retaining the 3D organization of the epigenome, we used fluorescence microscopy. Due to the resolution limit, conventional confocal systems are not able to unravel these interactions at the nanoscale. Therefore, we investigated whether the improved resolution obtained via 4× expansion microscopy could allow us to obtain insight into highly abundant chromatin readers and quantify them at a single-cell level (Figure 1). We refer to this method as Expansion Microscopy for Epigenetics or ExEpi

**Figure 2.**
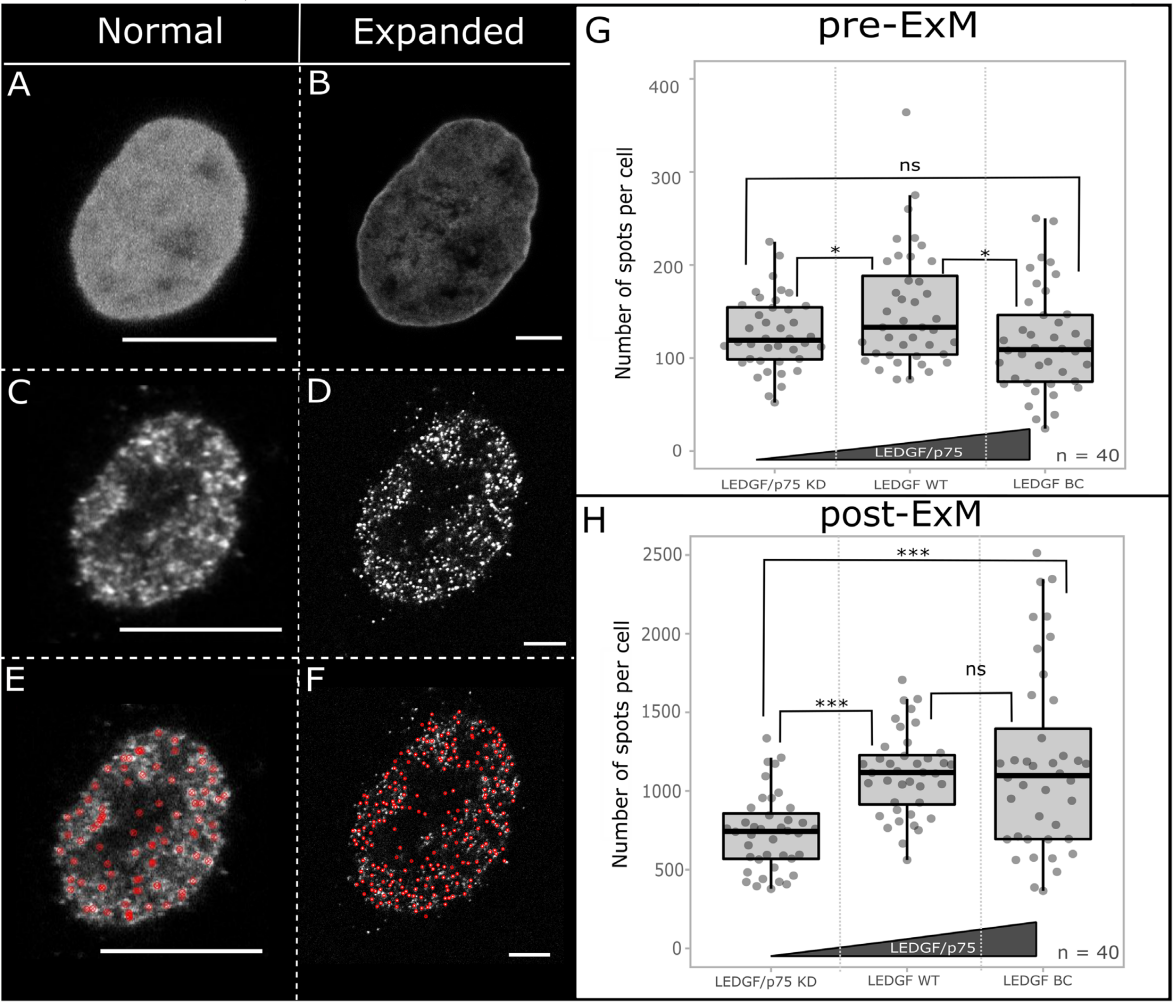
Imaging of the same HeLaP4 cell before (**A**, **C**, **E**) and after (**B**, **D**, **E**) expansion. Quantification of LEDGF in HeLaP4 cells with WT levels of LEDGF (p75 and p52) (LEDGF WT), with reduced p75 levels (LEDGF/p75 KD) and in LEDGF KD back complemented cells (LEDGF BC). (A, B) DAPI staining for segmentation of the nucleus and identification of cells pre- and post-expansion. Due to improved resolution after expansion, the DAPI staining appears less homogenous, corresponding with the distribution of the chromatin (30). (C, D) Immunostaining of LEDGF/p75 and p52 proteins before and after expansion; secondary antibody: GAM Alexa488 (4 μg/ml). (E, **F**) MATLAB routine output with red circles indicating what is counted as a spot. (**G**, **H**) Box plots showing quantification of counted spots both pre-ExM and post-ExM in HeLaP4 cells with varying LEDGF/p75 expression, where each grey dot represents one cell. The polyclonal nature of LEDGF BC cells results in a strong variation in expression levels post-ExM (H). The used primary antibody (2 μg/ml) targets both LEDGF/p75 and LEDGF/p52 proteins. Statistical analysis was performed by a One-way ANOVA: ns) non-significant; *P-value < 0.05; ***P-value <0.001; number of cells (*n*) = 40, with two different samples analyzed per condition. The presented images (A–F) are single optical sections. Scale bars: 10 μm (A–F). Details on used antibodies and dilutions can be found in SI Table 11.

To evaluate if expansion microscopy can yield the required resolution, we used HeLaP4 cell lines described by Gijsbers *et al.* (28) with LEDGF/p75 expression ranging from lower (knockdown) to higher levels (back complementation) when compared to wild type cells (LEDGF WT). First, the expression levels were quantified by regular immunofluorescence microscopy and an intensity-based analysis, using an antibody that detects both LEDGF/p75 and LEDGF/p52 (Supplementary Fig. 1). In the LEDGF/p75 knockdown cell-line (LEDGF/p75 KD) a ∼70% decrease in the fluorescence signal was measured when compared to WT cells whereas a ∼50% increase in the mean intensity was measured when LEDGF/p75 back complemented (LEDGF BC) cells were used. The variation in observed intensity in LEDGF BC cells reflects their polyclonal nature (Supplementary Table 1). These results were in line with a western blot analysis, showing a strong decrease (∼99.6%) in signal in LEDGF/p75 KD cells. In LEDGF BC cells, a ∼40% increase was observed when both LEDGF/p75 and LEDGF/p52 signals were taken into account (Supplementary Fig. 2). Next, we used an in-house written MATLAB routine to count the number of detected LEDGF (p75 and p52) spots in a single cell. When the same cell is measured pre- and post-expansion (Figure 2A, B), a clear difference in the number of spots is evidenced (pre-ExM = 51 spots; post-ExM = 1620 spots) due to the enhanced resolution after expansion (Figure 2C, D), enabling a more exact quantification of LEDGF/p75 and p52 (Figure 2E, F). This gain in resolution was also needed to discriminate the number of spots. Before expansion, it appeared like both LEDGF/p75 KD and LEDGF BC had a lower number of detected spots when compared to LEDGF WT (Figure 2G). However, in expanded cells, a significant drop in the number of counted spots was observed in LEDGF/p75 KD cells with a *P*-value <0.001 between different groups (Supplementary Tables 2 and 3, Supplementary Figure 3). These results indicate that an improved resolution enables a more precise representation of the amount of LEDGF present in each nucleus. Nevertheless, discriminating for single proteins with certainty is still not possible after expansion due to their small size (∼5–10 nm) (29) implying that the number of calculated spots is a relative value.

### Quantification of co-localization using ExEpi

**Figure 3.**
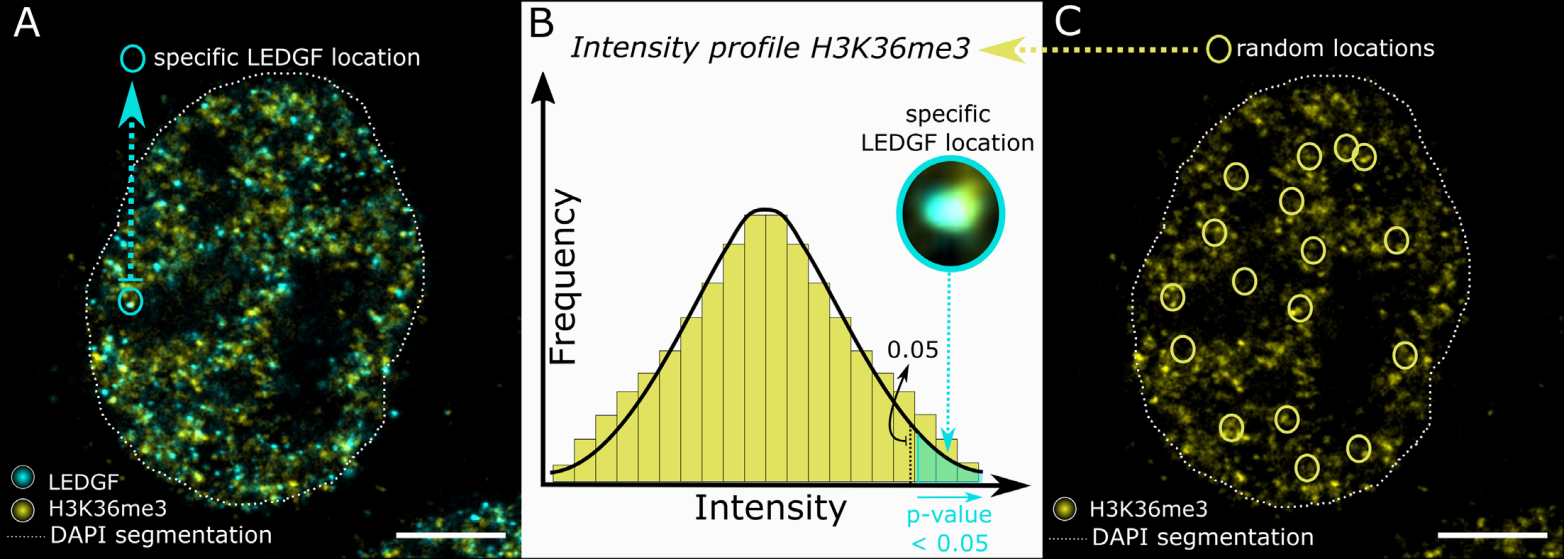
Schematic illustration of the concept of co-localization analysis. (**A**) Nuclear segmentation is indicated by the dotted white line. Inside the nucleus of a HeLaP4 cell, both the LEDGF protein (cyan) and H3K36me3 marker (yellow) are shown via immunostaining, with indication of the location of a specific LEDGF spot circled in cyan. (**B**) A zoom of this specific LEDGF location is shown and the corresponding H3K36me3 intensity at this exact location is plotted onto the overall intensity profile of H3K36me3 to calculate a *P*-value. If such a *P*-value is found to be lower than 0.05, co-localization is considered to occur. (**C**) Random locations (yellow circles) in the marker channel of the same cell as shown in (A) are used to generate a distribution of intensity values as shown in (B). The presented images (A, C) are single optical sections. Scale bars: 10 μm (A, C). Details on used antibodies and dilutions can be found in SI Table 11.

After demonstrating that expansion microscopy can quantify the overall number of protein spots in a single cell better than regular confocal imaging, we quantified the interaction of an epigenetic reader with a specific histone modification through co-localization based image analysis in expanded samples. For this, we acquired microscopy images in three different wavelength channels. A DAPI staining for the nucleus, a first immunostaining to detect the epigenetic reader and a second immunostaining for the specific epigenetic mark were utilized to assess co-localization (Supplementary Figure 4A). Briefly, the 3D location of epigenetic reader proteins is determined (Figure 3), using algorithms for single-molecule detection. However, localization of the H3K36me3 marker for example is less evident due to its heterogeneous distribution throughout the nucleus which results in strong intensity fluctuations, as already seen in previous STORM images (31). Therefore, co-localization of the epigenetic marker with the protein of interest is evaluated by comparing the fluorescence intensity of the marker at the protein location with the distribution of fluorescence intensity at random locations inside the nucleus (Figure 3B, C). When the intensity of the marker at a specific protein location is contrasted with this distribution, an empirical *P*-value can be calculated (Figure 3B) for this exact protein location. A protein is defined as co-localizing when a low *p*-value is found (*P*-value < 0.05) (Supplementary Fig. 4B, C) meaning both the protein and marker will co-exist in the same *x*, *y* and *z* position. To demonstrate more visually what type of overlap is characterized as co-localization, additional images are included in the Supplementary Information (Supplementary Figure 5A, B). Here, four different reader spots for both LEDGF and BRD4 proteins are highlighted with either (i) clear overlap with the corresponding histone modification, (ii) no overlap whatsoever or readers just on the edge of (iii) co-localization or (iv) no co-localization. Furthermore, the distance from each protein spot towards the edge of the nucleus was also calculated and used for spatial studies. As a first test to validate this type of quantification, we made use of dual color labelled HIV-1 viral particles in regular confocal microscopy. These express both Vpr-mCherry (RFP) and Vpr-eGFP and function as a positive control. When mCherry expression was localized and the corresponding intensity of eGFP expression at this position determined, a *P*-value <0.05 was estimated for 265 out of 287 particles, corresponding to a co-localization of 92% (Supplementary Figure 6). We assign the missing 8% co-localization to variations in expression of the fluorescent proteins.

### Co-localization of LEDGF with various epigenetic marks analyzed by ExEpi

**Figure 4.**
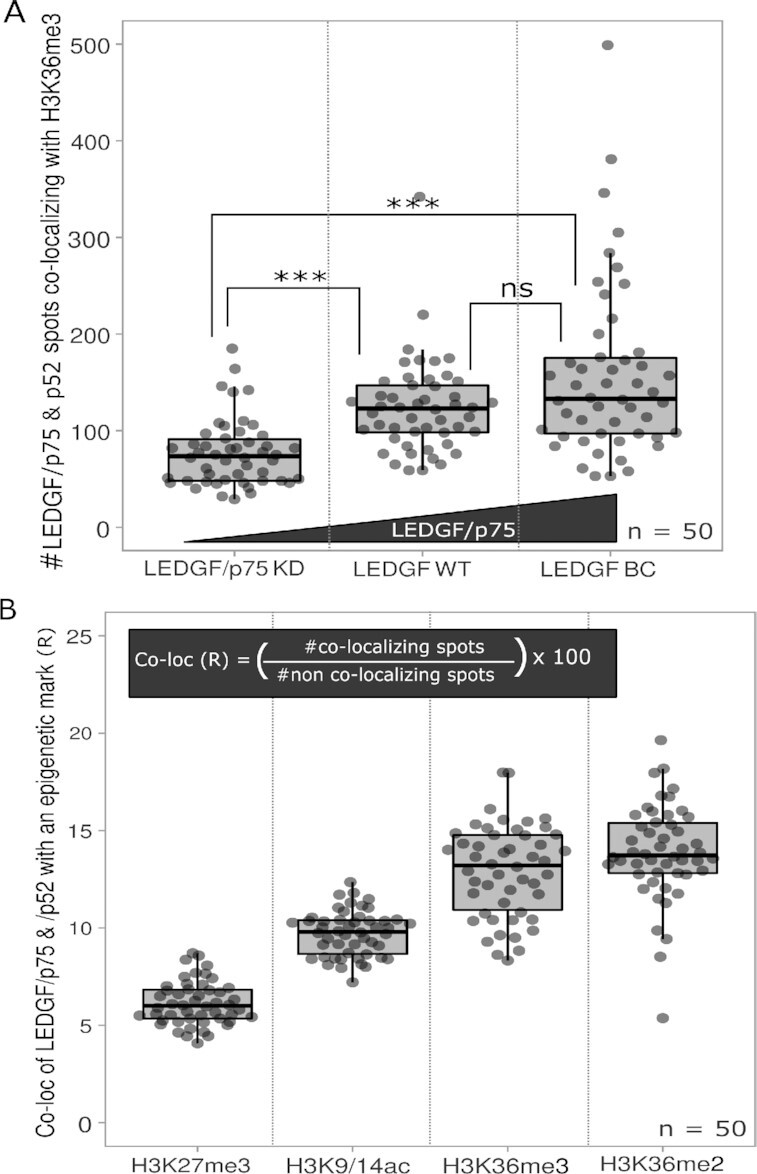
Co-localization of LEDGF/p75 and p52 with different epigenetic modifications. (**A**) Number of co-localizing LEDGF spots with H3K36me3 was measured in HeLaP4 cells (LEDGF WT – LEDGF/p75 KD – LEDF BC). (**B**) Co-localization ratio of LEDGF with H3K27me3 – H3K9/14ac – H3K36me3 – H3K36me2 marks. Statistical analysis was performed by a one-way ANOVA: (ns) non-significant; ***P-value < 0.001; number of cells (*n*) = 50 with each grey dot representing one cell and two different samples analyzed per condition. Co-localization analysis was performed on Z-stacks of expanded cells consisting of 9–11 Z-slices with a Z-step size of 0.2 μm.

After validating calculations in ExEpi, we investigated the distribution of LEDGF (p75 and p52) in relation to the H3K36me3 modification by using the previously mentioned LEDGF/p75 KD, LEDGF WT and LEDGF BC cells (Figure 4A, Supplementary Table 4). When the number of co-localizing spots was counted in LEDGF/p75 KD cells (77 ± 34 spots), a significant decrease (*P*-value < 0.001) was observed when compared to cells with WT LEDGF expression (124 ± 48 spots) whereas in LEDGF BC cells an increase in co-localizing spots was detected (157 ± 90 spots; *P*-value < 0.05) due to the over-expression of the protein. Next to testing cells with different expression levels of LEDGF/p75, co-localization of LEDGF/p75 and p52 was also studied for a range of different epigenetic modifications. Four different modifications were examined: H3K27me3, a marker for heterochromatin, and three markers for euchromatin: H3K9/14ac, H3K36me3 and H3K36me2 (Figure 4B, Supplementary Table 5). To normalize between different markers and get an idea about the ratio between bound and unbound LEDGF proteins, co-localization ratios (*R*) were calculated by dividing the number of co-localizing spots with the number of non-co-localizing spots (total number of spots minus co-localizing spots). An average co-localization ratio of 6.13 ± 1.12 was observed for H3K27me3, which is a modification known to repress gene expression since it is associated with densely packed chromatin hampering the binding of transcription factors (31). In contrast, modifications such as acetylation (H3K9/14ac) of histones relax the chromatin conformation and make it more accessible for protein binding. As such, an increase in the co-localization ratio (9.72 ± 1.1 6 R) with LEDGF/p75 and p52 was observed when compared to silent DNA (H3K27me3). Within this analysis, the highest co-localization was observed for epigenetic marks known to specifically interact with LEDGF like H3K36me3 (12.92 ± 2.35 R) and H3K36me2 (13.87 ± 2.48 R). These methylated marks are preferred binding sites of LEDGF (32) in comparison with the di-acetylation marker (H3K9/14ac). To gain additional insights in the epigenetic landscape, we also tried to measure the ratio of epigenetic marks occupied by LEDGF/p75 and p52. To quantify this, we reversed the analysis and recalculated co-localization ratios (Supplementary Figures 7–10 and Supplementary Tables 6–8).

### LEDGF antibody specificity and background investigation of ExEpi

A western blot was performed on HEK293T WT and HEK239T LEDGF/p75 knock out cells to check the performance of the primary antibody for LEDGF detection (Supplementary Figures 11 and 12) which underlines the importance of working with specific antibodies when interpreting ExEpi results. In addition to the specificity of the LEDGF antibodies, the robustness of ExEpi itself was also examined. To get an idea about the ratio of false positives ExEpi detects, which is the overlapping signal between protein and marker when there is no actual interaction, we rotated the images of one channel over different angles to force a mismatch in overlap between protein and marker and as such generate a certain number of random co-localization events to function as a negative control (33). For this particular analysis, zooms of the cell were analyzed where both the marker and reader were detected (Supplementary Figure 13A). The co-localization analysis was run on images of LEDGF WT cells with H3K36me3 staining. Here we found 5.16 ± 2.52 R co-localization (Supplementary Fig. 13B) for a 180° rotation of the LEDGF channel compared to 11.84 ± 3.91 R co-localization when there was no rotation (0°), showing indeed a certain level of random co-localization that is not attributed to biological interaction. We refer to this number as background.

### Co-localization of BRD4 with various epigenetic marks

**Figure 5.**
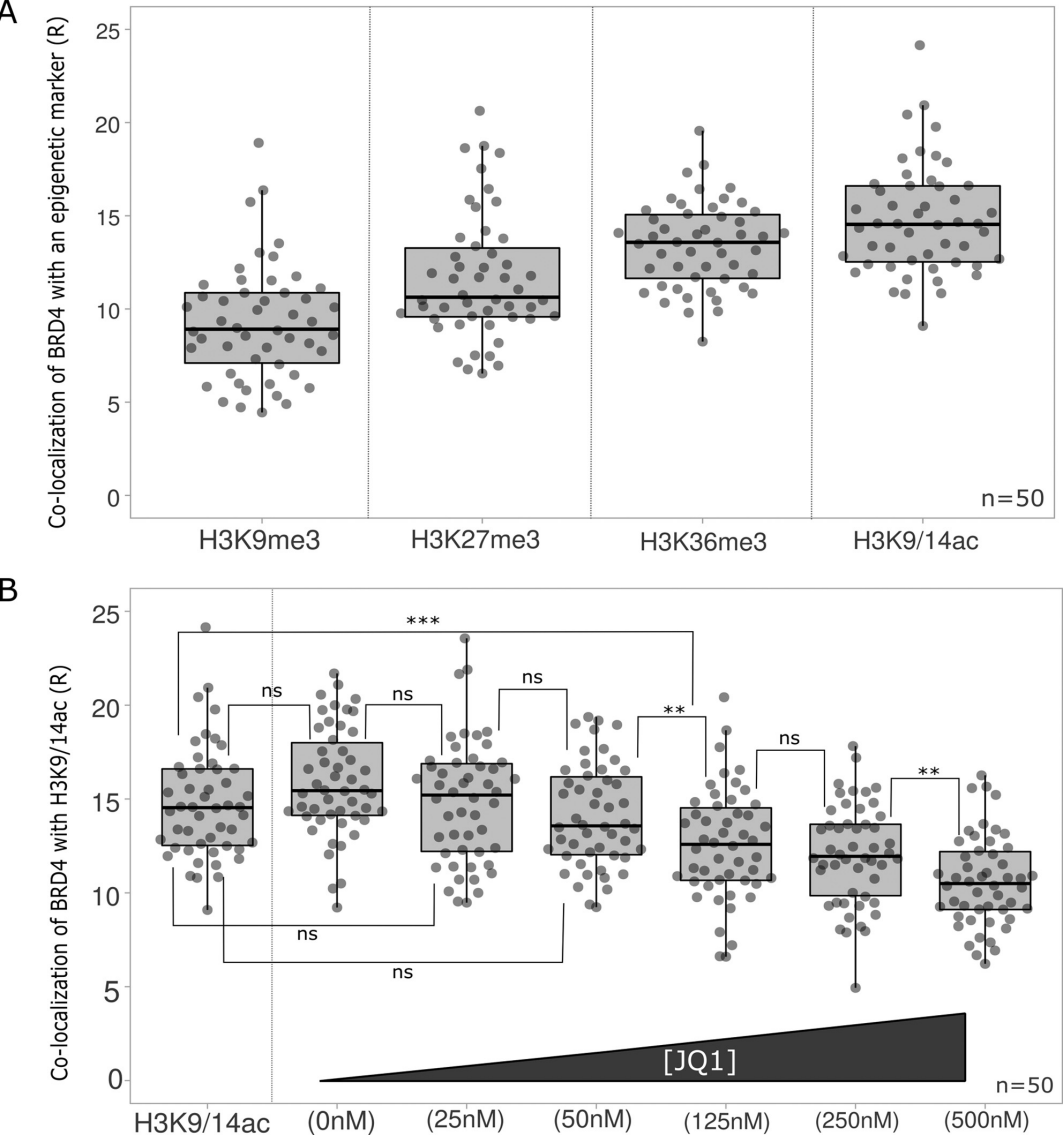
Co-localization of BRD4 with different epigenetic modifications and inhibition by JQ1. (**A**) Co-localization of BRD4 with H3K9me3, H3K27me3, H3K36me3 or H3K9/14ac marks. Co-localization was measured in HeLaP4 cells. (**B**) Co-localization of BRD4 with H3K9/14ac in the presence of an increasing JQ1 concentration: 0, 25, 50, 125, 250 and 500 nM. Statistical analysis was performed by a one-way ANOVA: (ns) non-significant; **P-value < 0.01; ***P-value < 0.001; number of cells (*n*) = 50 with each grey dot representing one cell and two different samples analyzed per condition. Co-localization analysis was performed on Z-stacks of expanded cells consisting of 9–11 Z-slices with a Z-step size of 0.2 μm.

We also used ExEpi to study the BRD4 distribution with respect to two heterochromatin markers, H3K9me3 and H3K27me3, and two euchromatin markers, H3K/14ac and H3K36me3 (Figure 5A). Since acquired ratios are not absolute numbers, these values can only be compared within the BRD4 experiment itself and not with earlier calculated LEDGF ratios. Nevertheless, a similar trend can still be observed, showing lowest co-localization with markers for silent chromatin: 9.27 ± 3.09 R with H3K9me3 and 11.67 ± 3.38 R for H3K27me3 (Supplementary Table 9). Co-localization with an euchromatin marker like acetylated H3K9 and H3K14 showed the highest ratio (14.75 ± 2.96 R), in agreement with the known characteristics of BRD4 as an acetylation reader (26). On the other hand, tri-methylated H3K36, which is another marker for euchromatin, also revealed co-localization (13.42 ± 2.32 R). In addition, we investigated whether BRD4 condensates could be distinguished with our ExEpi approach since many studies on BRD4 phase separation and focus formation have emerged over time (34). Since our research is not particularly aimed at detection of BRD4 condensates, preliminary findings can be found in the supplementary information (Supplementary Figures 14–16).

### Use of ExEpi in epigenetic drug discovery

Finally, we examined the effect of the known BET inhibitor JQ1 on the co-localization of BRD4 with the di-acetylation marker (H3K9/14ac) (Figure 5B). JQ1 is known to interact with the acetyl-lysine binding pocket of BRD4 by mimicking the shape of acetylated lysine residues(25), hindering the protein from binding to the chromatin. Since JQ1 is dissolved in DMSO, we first verified whether addition of DMSO interferes with co-localization. No significant differences (*p*-value > 0.05) were observed for the co-localization with H3K9/14ac in the absence of DMSO (14.75 ± 2.96 R) compared to 0.3% DMSO (15.88 ± 2.89 R). A competitive inhibition was observed when gradually increasing the JQ1 concentration from 0 to 500 nM, reducing co-localization with H3K9/14ac by a third (0 nM = 15.88 ± 2.89 R; 500 nM = 10.62 ± 2.41 R) (Supplementary Table 10). From 125 nM onwards, there is a significant reduction (*P*-value < 0.001) in co-localization with the di-acetylation marker and an IC_50_ of 137 nM was calculated based on these data (Supplementary Figure 17).

### Spatial organization of co-localization of LEDGF and BRD4 with their respective histone marks

**Figure 6.**
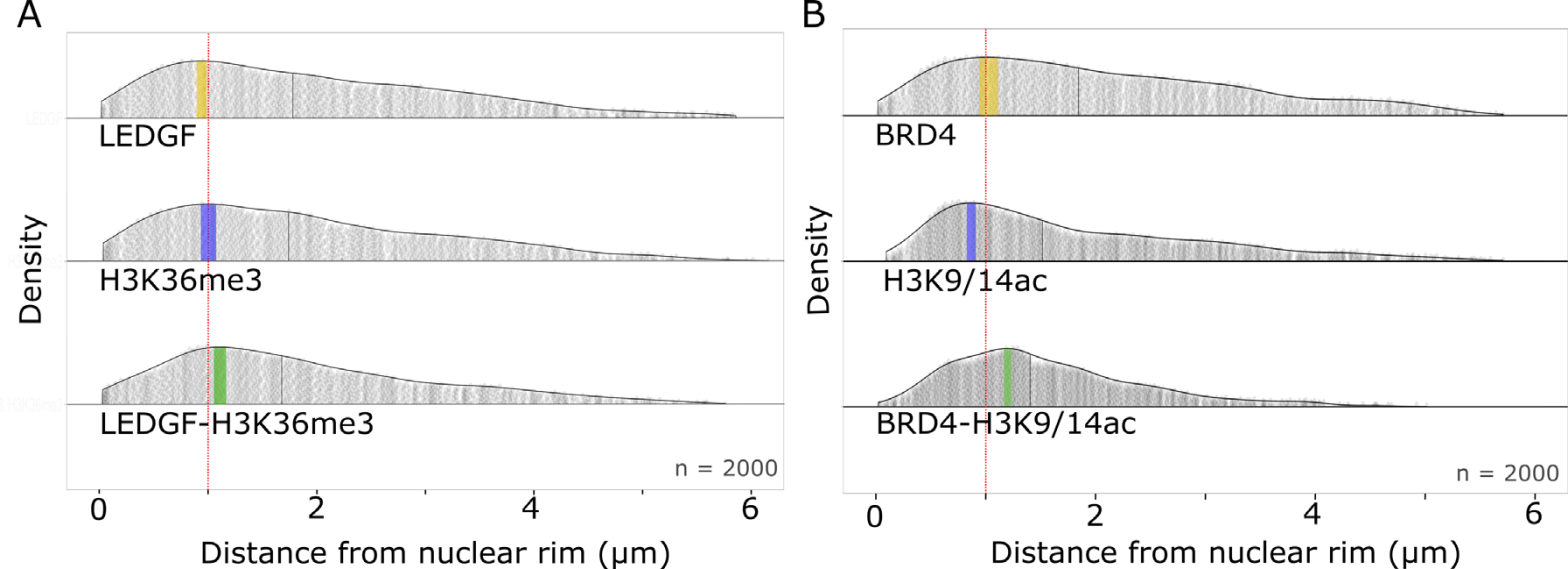
Spatial organization of epigenetic readers and histone marks in HeLaP4 cells. (**A**) Density plots for the distance (μm) of LEDGF, H3K36me3 and co-localizing spots to the nuclear rim. The median distance is presented as a thin black line inside the density plot: LEDGF = 1.75 μm; H3K36me3 = 1.71 μm; LEDGF – H3K36me3 = 1.64 μm. The region with the highest density is colored for each plot: LEDGF in yellow ∼0.9 μm; H3K36me3 in blue ∼1.0 μm; LEDGF – H3K36me3 in green ∼1.15 μm. (**B**) Density plots for the distance (μm) of BRD4, H3K9/14ac and co-localizing spots to the nuclear rim. The median distance is presented as a thin black line inside the density plot: BRD4 = 1.80 μm; H3K9/14ac = 1.48 μm; BRD4 – H3K9/14ac = 1.39 μm. The region with the highest density is colored for each plot: BRD4 in yellow ∼ 1.1 μm; H3K9/14ac in blue ∼ 0.8 μm; BRD4 – H3K9/14ac in green ∼ 1.25 μm. The red dotted line inside the density plot represents a distance of 1.0 μm. The average radius of a HeLaP4 nucleus is 6.00 ± 0.69 μm. Number of analyzed spots (*n*) = 2000 with each grey dot representing one spot (protein, marker or co-localizing protein). The spatial analysis was performed on Z-stacks of expanded cells consisting of 9–11 Z-slices with a Z-step size of 0.2 μm.

ExEpi allows to investigate not only the co-localization of epigenetic readers and histone marks, but also the distribution of these co-localizing readers within the nuclear space. Since ExEpi relies on fluorescence imaging, spatial information inside the nucleus is retained in contrast to ChIP for instance. We calculated the distance from the nuclear rim, determined by DAPI staining, of co-localizing BRD4 and LEDGF spots and divided that distance by an average expansion factor of ∼3.5 to obtain actual distances within the nucleus. We focused for each protein on the histone mark with the highest co-localization ratio. As such, distances were determined for LEDGF, H3K36me3 and LEDGF co-localizing with H3K36me3 (Figure 6A) or BRD4, H3K9/14ac and BRD4 co-localizing with H3K9/14ac (Figure 6B). In panel A LEDGF proteins show a broad distribution with a maximal density in the nuclear periphery at ∼0.9 μm distance from the nuclear rim while H3K36me3 displays a similar distribution with a maximum at ∼1.0 μm distance. LEDGF co-localizes preferentially with H3K36me3 at ∼ 1.15 μm. In panel B, BRD4 proteins are found with a wide distribution but a maximum at ∼1.1 μm distance while H3K9/14ac is closer to the nuclear rim at ∼0.8 μm distance. BRD4 co-localizes with H3K9/14ac deeper into the nucleus at ∼1.25 μm. As a control, the distance of the heterochromatin mark, H3K9me3, was also analyzed and this marker is located in the nuclear rim between ∼0.0 and 0.2 μm (Supplementary Figure 18).

## DISCUSSION

Here, we present a novel co-localization method to study interactions of chromatin readers with specific epigenetic modifications. To retain spatial information in single cells, co-localization studies are done by combining immunostaining with expansion microscopy and referred to as ExEpi. We observed improved detection of chromatin readers by comparing counted LEDGF spots (p75 and p52) before and after expansion, showing an increase in counted molecules post-expansion due to de-crowding (35). Because of this subsequent gain in resolution, we could also highlight differences in LEDGF/p75 expression levels in cells depleted for LEDGF/p75 (28) (Figure 2). This difference was not observed in pre-expansion images, demonstrating the need for expansion. Although the expansion process makes a more accurate detection of the protein of interest possible, the defined spots visible after expansion do not necessarily represent single proteins since the size of the proteins covered with a primary and secondary antibody is ∼25 nm (29) and thus, resolution is still limiting after expansion. Therefore, counting protein spots does provide relative and not absolute numbers.

We used ExEpi to investigate the co-localization of LEDGF (Figure 4) and BRD4 (Figure 5) proteins with a range of epigenetic markers representing either hetero- or euchromatin. LEDGF (p75 and 52) interaction increased when moving from heterochromatin (H3K27me3) towards open chromatin and a clear preference was observed for di- and tri-methylated H3K36 over histone acetylation (H3K9/14ac) which is in line with previous studies (32, 36). The extent of co-localization with the preferred epigenetic mark H3K36me3 was dependent on the expression level of LEDGF/p75 indicative of a binding equilibrium that is not saturated neither by biology, nor by ExEpi.

To determine background interactions, a LEDGF/p75-specific knock out cell line was analyzed (SI Figures 11 and 12). Because the used primary antibody targets both LEDGF/p75 and p52, LEDGF/p52 is still detected in the knockout cells, confounding the determination of background co-localization. Therefore, we opted for a more methodological approach as a negative control. By performing a 180° rotation of all images in one channel, the detected spots were no longer at their original location, hampering correct co-localization. Nevertheless, a co-localization of 5.6 ± 1.1 R between LEDGF and H3K36me3 was measured after image rotation, revealing detection of a certain number of random co-localization events or false positives.

It should be noted that a conventional co-localization method such as the Pearson's correlation coefficient (PCC) was not applied here. Although this coefficient is useful when working with non-equal level of signals, it depends on a linear relation between these signals over the entire data set or image and is therefore challenging to interpret when only looking at the overlap between specific molecules (33). When a PCC analysis (Supplementary Figure 19) was performed, a low correlation with an average PCC of 0.13 ± 0.04 was calculated for the known interaction between LEDGF and H3K36me3 and a 180° rotation of the LEDGF channel in the H3K36me3 showed no correlation (average PCC = 0.02 ± 0.03). When the interaction of LEDGF with another epigenetic mark was investigated (H3K9/14ac; 0.12 ± 0.03) no significant difference was found when compared to H3K36me3 (*P*-value > 0.05). We attribute these overall low PCC values to the intensity fluctuations in the marker channel, resulting in not well-defined molecules, making it challenging to define background and perform a correct correlation. Hence small differences between readers and markers cannot be detected with the known co-localization methods and alternative calculations as proposed in this paper are required to achieve high sensitivity.

Next, the co-localization of BRD4 with several histone modifications was analyzed. Similar to LEDGF results, co-localization ratios were obtained for two heterochromatin markers (H3K9me3 and H3K27me3) with the lowest ratio of 9.27 ± 3.09 for H3K9me3, which is consistent with previous mass spectrometry research (16). Within this assay, distinct co-localization was observed for H3K9/14ac (14.75 ± 2.96 R) confirming that BRD4 is a reader with a strong affinity for acetylated histones (37). Addition of JQ1, a BET inhibitor that mimics the shape of acetyl-lysine residues and blocks the bromodomain by binding in the acetyl-lysine pocket of BRD4 (25), resulted in a clear concentration-dependent decrease in co-localization with H3K9/14ac. The analyzed JQ1 concentrations were in line with earlier research where an IC_50_ of 33 nM for the first bromodomain of BRD4 and 77 nM for the second domain were measured by Alpha-screen titrations (25). Although a complete inhibition in co-localization was not accomplished, we calculated an IC_50_ of 137 nM. This result was similar to previous research when JQ1 was used to examine the function of BET proteins in the viral replication cycle of the Murine Leukemia Virus (MLV) (38). Based on a MLV-luciferase assay, an IC_50_ of 122 nM was determined, proving specific bromodomain inhibitors like JQ1 block viral replication. As such, ExEpi may also find use in epigenetic drug discovery.

The advantage of ExEpi is the possibility to obtain spatial information. We acquired information about LEDGF and BRD4 spots co-localizing with their preferred euchromatin marker, H3K36me3 and H3K9/14ac, respectively (Figure 6). For each reader, the distance from the co-localizing spot to the nuclear rim was obtained. Of note the preferential position of both epigenetic readers when co-localizing with their histone marker was shifted more towards the center of the nucleus than the preferred location for protein and mark, separately. Interestingly, the detected location of the histon marks corresponds with previous research (Supplementary Fig. 18). The heterochromatin mark, H3K9me3, localizes nearby the nuclear rim (∼0.00–0.20 μm) which is in line with literature, although it can also be found closer the center of the nucleus, in the context of highly repetitive heterochromatin (39). The di-acetylation marker (H3K9/14ac) was located more towards the center of the nucleus (∼0.8 μm) when compared to H3K9me3. Nevertheless, H3K9/14ac is still located closer to the nuclear rim than the euchromatin marker H3K36me3 (∼1.0 μm) and therefore present in an area between H3K9me3 and H3K36me3, similar to the location of H3K27me3, as previously demonstrated in both Drosophila and mammalian cells (40, 41). When looking into the location of the epigenetic readers, we find LEDGF in the nuclear periphery at a distance around ∼ 0.9 μm from the nuclear rim whereas BRD4 can be found at a slightly deeper location at ∼1.1 μm. Both are transcriptional co-activators (21, 23) and transcriptionally active chromatin is conventionally found deeper inside the nucleus (41), which also may explain the shift in location when it is bound to acetylated chromatin (∼1.2 μm). Remarkably, when LEDGF co-localizes with H3K36me3, it can be found at a preferential distance between ∼ 1.10 – 1.20 μm which correlates with earlier research that has shown that the average penetration depth of HIV-1 pre-integration complexes carrying YFP labeled HIV-1 integrase is 1.4 ± 0.4 μm in HeLaP4 cells (42). These findings corroborate the tight relation between HIV-1 pre-integration complexes targeted by LEDGF/p75 to H3K36me3 and can explain the preferential area in the nucleus where HIV-1 integrates. Integration of HIV-1 in the nuclear periphery has been demonstrated in different cells (43–45).

To conclude, ExEpi enables investigation of the epigenetic landscape within a single cell, making straightforward analysis of cell to cell variability possible. Here, we mainly focus on co-localization ratios between different epigenetic readers and histone modifications. However, because spatial information is retained, in contrast to other methods like ChIP-Seq and MS, ExEpi was also used to visualize the exact nuclear location of epigenetic readers and epigenetic markers. This is useful since the spatial arrangement of chromatin and histone PTM’s can vary between different cell types and as such influence nuclear functions as has been demonstrated before in rod photoreceptor cells where for example the location of the H3K9me3 PTM will shift towards the center of the nucleus to function as a light collecting lens (46). In addition, because ExM can be used to study tissue sections (18, 47), ExEpi also has the potential to enable single-cell studies of heterogeneous tumor tissues since the preserved spatial arrangement will add another dimension to the single-cell analysis and as such unravel how cells are organized and interact across the tissue landscape. Although information about the genomic location of epigenetic readers and histone PTMs in complex tissues can already be achieved by scChIP-seq (13) or scCUT&Tag (14) methodologies, direct interactions between a reader and histone PTM are not shown. Furthermore, we expect that ExEpi could be combined in the future with fluorescent *in situ* hybridization (FISH) of RNA and DNA (35, 48), to obtain a complete read-out of a single cell by linking transcriptomics, genomics and epigenetics, since a recent study showed the use of ExM to quantify histone modifications at a single-gene level (49). In addition, the results described in this research are not yet at the molecular level since resolution is still limited. Therefore, one cannot be completely sure that the obtained co-localization ratios reflect actual interactions or rather a proximity of the molecules of interest. Nevertheless, obtained results highlight the existence of possible spatial relationships since the data are in line with already known preferred interactions like the one of BRD4 with histone acetylation (26) and LEDGF with H3K36me3 (21). In the future, implementation of higher expansion factors through 10-fold expansion (50) or iterative expansion (51) could be helpful to achieve a ∼25 nm resolution and uncover the complete cellular landscape at the molecular level.

## Supplementary Material

gkac521_Supplemental_File

## Data Availability

MATLAB codes for image processing and co-localization analysis are available at GitHub (https://github.com/BorisLouis/Colocalization). Other data related to this research can be requested via the corresponding authors.

## References

[1] Cavalli G. , Heard E. Advances in epigenetics link genetics to the environment and disease. Nature. 2019; 571:489–499. 31341302 10.1038/s41586-019-1411-0

[2] Flavahan W.A. , Gaskell E., Bernstein B.E. Epigenetic plasticity and the hallmarks of cancer. Science. 2017; 357:eaal2380. 28729483 10.1126/science.aal2380 PMC5940341

[3] Zhang P. , Torres K., Liu X., Liu C., Pollock R.E. An overview of chromatin-regulating proteins in cells. Curr. Protein Pept. Sci. 2016; 17:401–410. 26796306 10.2174/1389203717666160122120310 PMC4932839

[4] Mirabella A.C. , Foster B.M., Bartke T. Chromatin deregulation in disease. Chromosoma. 2016; 125:75–93. 26188466 10.1007/s00412-015-0530-0 PMC4761009

[5] Audia J.E. , Campbell R.M. Histone modifications and cancer. Cold Spring Harb. Perspect. Biol. 2016; 8:a019521. 27037415 10.1101/cshperspect.a019521 PMC4817802

[6] Zentner G.E. , Henikoff S. Regulation of nucleosome dynamics by histone modifications. Nat. Struct. Mol. Biol. 2013; 20:259–266. 23463310 10.1038/nsmb.2470

[7] Cai Y. , Zhang Y., Loh Y.P., Tng J.Q., Lim M.C., Cao Z., Raju A., Lieberman Aiden E., Li S., Manikandan L. et al. H3K27me3-rich genomic regions can function as silencers to repress gene expression via chromatin interactions. Nat. Commun. 2021; 12:719. 33514712 10.1038/s41467-021-20940-y PMC7846766

[8] Wagner E.J. , Carpenter P.B. Understanding the language of Lys36 methylation at histone H3. Nat. Rev. Mol. Cell Biol. 2012; 13:115–126. 22266761 10.1038/nrm3274 PMC3969746

[9] Musselman C.A. , Lalonde M.-E., Côté J., Kutateladze T.G. Perceiving the epigenetic landscape through histone readers. Nat. Struct. Mol. Biol. 2012; 19:1218–1227. 23211769 10.1038/nsmb.2436 PMC3645987

[10] Wang G.G. , Song J., Wang Z., Dormann H.L., Casadio F., Li H., Luo J.-L., Patel D.J., Allis C.D. Haematopoietic malignancies caused by dysregulation of a chromatin-binding PHD finger. Nature. 2009; 459:847–851. 19430464 10.1038/nature08036 PMC2697266

[11] Gilmour D.S. , Lis J.T. Detecting protein-DNA interactions in vivo: distribution of RNA polymerase on specific bacterial genes. Proc. Natl. Acad. Sci. U.S.A. 1984; 81:4275–4279. 6379641 10.1073/pnas.81.14.4275 PMC345570

[12] O’Geen H. , Echipare L., Farnham P.J. Using ChIP-Seq technology to generate high-resolution profiles of histone modifications. Methods Mol. Biol. Clifton NJ. 2011; 791:265–286. 10.1007/978-1-61779-316-5_20 PMC4151291 21913086

[13] Grosselin K. , Durand A., Marsolier J., Poitou A., Marangoni E., Nemati F., Dahmani A., Lameiras S., Reyal F., Frenoy O. et al. High-throughput single-cell ChIP-seq identifies heterogeneity of chromatin states in breast cancer. Nat. Genet. 2019; 51:1060–1066. 31152164 10.1038/s41588-019-0424-9

[14] Bartosovic M. , Kabbe M., Castelo-Branco G. Single-cell CUT&Tag profiles histone modifications and transcription factors in complex tissues. Nat. Biotechnol. 2021; 39:825–835. 33846645 10.1038/s41587-021-00869-9 PMC7611252

[15] Verhelst S. , De Clerck L., Willems S., Van Puyvelde B., Daled S., Deforce D., Dhaenens M. Comprehensive histone epigenetics: a mass spectrometry based screening assay to measure epigenetic toxicity. MethodsX. 2020; 7:101055. 32995308 10.1016/j.mex.2020.101055 PMC7508989

[16] Villaseñor R. , Pfaendler R., Ambrosi C., Butz S., Giuliani S., Bryan E., Sheahan T.W., Gable A.L., Schmolka N., Manzo M. et al. ChromID identifies the protein interactome at chromatin marks. Nat. Biotechnol. 2020; 38:728–736. 32123383 10.1038/s41587-020-0434-2 PMC7289633

[17] Xu J. , Liu Y. A guide to visualizing the spatial epigenome with super-resolution microscopy. FEBS J. 2019; 286:3095–3109. 31127980 10.1111/febs.14938 PMC6699889

[18] Chen F. , Tillberg P.W., Boyden E.S. Expansion microscopy. Science. 2015; 347:543–548. 25592419 10.1126/science.1260088 PMC4312537

[19] Chozinski T.J. , Halpern A.R., Okawa H., Kim H.-J., Tremel G.J., Wong R.O.L., Vaughan J.C. Expansion microscopy with conventional antibodies and fluorescent proteins. Nat. Methods. 2016; 13:485–488. 27064647 10.1038/nmeth.3833 PMC4929147

[20] Vanheusden M. , Vitale R., Camacho R., Janssen K.P.F., Acke A., Rocha S., Hofkens J. Fluorescence photobleaching as an intrinsic tool to quantify the 3D expansion factor of biological samples in expansion microscopy. ACS Omega. 2020; 5:6792–6799. 32258914 10.1021/acsomega.0c00118 PMC7114699

[21] Blokken J. , De Rijck J., Christ F., Debyser Z. Protein–protein and protein–chromatin interactions of LEDGF/p75 as novel drug targets. Drug Discov. Today Technol. 2017; 24:25–31. 29233296 10.1016/j.ddtec.2017.11.002

[22] Singh D.P. , Kimura A., Chylack L.T., Shinohara T. Lens epithelium-derived growth factor (LEDGF/p75) and p52 are derived from a single gene by alternative splicing. Gene. 2000; 242:265–273. 10721720 10.1016/s0378-1119(99)00506-5

[23] Donati B. , Lorenzini E., Ciarrocchi A. BRD4 and cancer: going beyond transcriptional regulation. Mol. Cancer. 2018; 17:164. 30466442 10.1186/s12943-018-0915-9 PMC6251205

[24] Lu L. , Chen Z., Lin X., Tian L., Su Q., An P., Li W., Wu Y., Du J., Shan H. et al. Inhibition of BRD4 suppresses the malignancy of breast cancer cells via regulation of Snail. Cell Death Differ. 2020; 27:255–268. 31114028 10.1038/s41418-019-0353-2 PMC7205888

[25] Filippakopoulos P. , Qi J., Picaud S., Shen Y., Smith W.B., Fedorov O., Morse E.M., Keates T., Hickman T.T., Felletar I. et al. Selective inhibition of BET bromodomains. Nature. 2010; 468:1067–1073. 20871596 10.1038/nature09504 PMC3010259

[26] Dey A. , Chitsaz F., Abbasi A., Misteli T., Ozato K. The double bromodomain protein Brd4 binds to acetylated chromatin during interphase and mitosis. Proc. Natl. Acad. Sci. U.S.A. 2003; 100:8758–8763. 12840145 10.1073/pnas.1433065100 PMC166386

[27] Maddon P.J. , Dalgleish A.G., McDougal J.S., Clapham P.R., Weiss R.A., Axel R. The T4 gene encodes the AIDS virus receptor and is expressed in the immune system and the brain. Cell. 1986; 47:333–348. 3094962 10.1016/0092-8674(86)90590-8

[28] Gijsbers R. , Ronen K., Vets S., Malani N., De Rijck J., McNeely M., Bushman F.D., Debyser Z. LEDGF hybrids efficiently retarget lentiviral integration into heterochromatin. Mol. Ther. 2010; 18:552–560. 20195265 10.1038/mt.2010.36 PMC2839429

[29] Erickson H.P. Size and shape of protein molecules at the nanometer level determined by sedimentation, gel filtration, and electron microscopy. Biol. Proced. Online. 2009; 11:32–51. 19495910 10.1007/s12575-009-9008-x PMC3055910

[30] Szczurek A.T. , Prakash K., Lee H.-K., Żurek-Biesiada D.J., Best G., Hagmann M., Dobrucki J.W., Cremer C., Birk U. Single molecule localization microscopy of the distribution of chromatin using Hoechst and DAPI fluorescent probes. Nucleus. 2014; 5:331–340. 25482122 10.4161/nucl.29564 PMC4152347

[31] Xu J. , Ma H., Jin J., Uttam S., Fu R., Huang Y., Liu Y. Super-resolution imaging of higher-order chromatin structures at different epigenomic states in single mammalian cells. Cell Rep. 2018; 24:873–882. 30044984 10.1016/j.celrep.2018.06.085 PMC6154382

[32] LeRoy G. , Oksuz O., Descostes N., Aoi Y., Ganai R.A., Kara H.O., Yu J.-R., Lee C.-H., Stafford J., Shilatifard A. et al. LEDGF and HDGF2 relieve the nucleosome-induced barrier to transcription in differentiated cells. Sci. Adv. 2019; 5:eaay3068. 31616795 10.1126/sciadv.aay3068 PMC6774727

[33] Dunn K.W. , Kamocka M.M., McDonald J.H. A practical guide to evaluating colocalization in biological microscopy. Am. J. Physiol. - Cell Physiol. 2011; 300:C723–C742. 21209361 10.1152/ajpcell.00462.2010 PMC3074624

[34] Sabari B.R. , Dall’Agnese A., Boija A., Klein I.A., Coffey E.L., Shrinivas K., Abraham B.J., Hannett N.M., Zamudio A.V., Manteiga J.C. et al. Coactivator condensation at super-enhancers links phase separation and gene control. Science. 2018; 361:eaar3958. 29930091 10.1126/science.aar3958 PMC6092193

[35] Chen F. , Wassie A.T., Cote A.J., Sinha A., Alon S., Asano S., Daugharthy E.R., Chang J.-B., Marblestone A., Church G.M. et al. Nanoscale imaging of RNA with expansion microscopy. Nat Meth. 2016; 13:679–684. 10.1038/nmeth.3899 PMC4965288 27376770

[36] Daugaard M. , Baude A., Fugger K., Povlsen L.K., Beck H., Sørensen C.S., Petersen N.H.T., Sorensen P.H.B., Lukas C., Bartek J. et al. LEDGF (p75) promotes DNA-end resection and homologous recombination. Nat. Struct. Mol. Biol. 2012; 19:803–810. 22773103 10.1038/nsmb.2314

[37] Filippakopoulos P. , Picaud S., Mangos M., Keates T., Lambert J.-P., Barsyte-Lovejoy D., Felletar I., Volkmer R., Müller S., Pawson T. et al. Histone recognition and large-scale structural analysis of the human bromodomain family. Cell. 2012; 149:214–231. 22464331 10.1016/j.cell.2012.02.013 PMC3326523

[38] De Rijck J. , de Kogel C., Demeulemeester J., Vets S., El Ashkar S., Malani N., Bushman F.D., Landuyt B., Husson S.J., Busschots K. et al. The BET family of proteins targets moloney murine leukemia virus integration near transcription start sites. Cell Rep. 2013; 5:886–894. 24183673 10.1016/j.celrep.2013.09.040 PMC4197836

[39] Smith C.L. , Poleshko A., Epstein J.A. The nuclear periphery is a scaffold for tissue-specific enhancers. Nucleic Acids Res. 2021; 49:6181–6195. 34023908 10.1093/nar/gkab392 PMC8216274

[40] Llorens-Giralt P. , Camilleri-Robles C., Corominas M., Climent-Cantó P. Chromatin organization and function in Drosophila. Cells. 2021; 10:2362. 34572010 10.3390/cells10092362 PMC8465611

[41] Penagos-Puig A. , Furlan-Magaril M. Heterochromatin as an important driver of genome organization. Front. Cell Dev. Biol. 2020; 8:982. 10.3389/fcell.2020.579137 PMC7530337 33072761

[42] Burdick R.C. , Delviks-Frankenberry K.A., Chen J., Janaka S.K., Sastri J., Hu W.-S., Pathak V.K. Dynamics and regulation of nuclear import and nuclear movements of HIV-1 complexes. PLoS Pathog. 2017; 13:e1006570. 28827840 10.1371/journal.ppat.1006570 PMC5578721

[43] Dieudonné M. , Maiuri P., Biancotto C., Knezevich A., Kula A., Lusic M., Marcello A. Transcriptional competence of the integrated HIV-1 provirus at the nuclear periphery. EMBO J. 2009; 28:2231–2243. 19478796 10.1038/emboj.2009.141 PMC2726691

[44] Vranckx L.S. , Demeulemeester J., Saleh S., Boll A., Vansant G., Schrijvers R., Weydert C., Battivelli E., Verdin E., Cereseto A. et al. LEDGIN-mediated inhibition of integrase–LEDGF/p75 interaction reduces reactivation of residual latent HIV. EBioMedicine. 2016; 8:248–264. 27428435 10.1016/j.ebiom.2016.04.039 PMC4919729

[45] Marini B. , Kertesz-Farkas A., Ali H., Lucic B., Lisek K., Manganaro L., Pongor S., Luzzati R., Recchia A., Mavilio F. et al. Nuclear architecture dictates HIV-1 integration site selection. Nature. 2015; 521:227–231. 25731161 10.1038/nature14226

[46] Solovei I. , Kreysing M., Lanctôt C., Kösem S., Peichl L., Cremer T., Guck J., Joffe B. Nuclear architecture of rod photoreceptor cells adapts to vision in mammalian evolution. Cell. 2009; 137:356–368. 19379699 10.1016/j.cell.2009.01.052

[47] Wassie A.T. , Zhao Y., Boyden E.S. Expansion microscopy: principles and uses in biological research. Nat. Methods. 2019; 16:33–41. 30573813 10.1038/s41592-018-0219-4 PMC6373868

[48] Wen G. , Vanheusden M., Leen V., Rohand T., Vandereyken K., Voet T., Hofkens J. 2021) A universal labeling strategy for nucleic acids in expansion microscopy. J. Am. Chem. Soc. 143:13782–13789. 34424689 10.1021/jacs.1c05931

[49] Woodworth M.A. , Ng K.K.H., Halpern A.R., Pease N.A., Nguyen P.H.B., Kueh H.Y., Vaughan J.C. Multiplexed single-cell profiling of chromatin states at genomic loci by expansion microscopy. Nucleic Acids Res. 2021; 49:e82. 34048564 10.1093/nar/gkab423 PMC8373070

[50] Truckenbrodt S. , Maidorn M., Crzan D., Wildhagen H., Kabatas S., Rizzoli S.O. X10 expansion microscopy enables 25-nm resolution on conventional microscopes. EMBO Rep. 2018; 19:e45836. 29987134 10.15252/embr.201845836 PMC6123658

[51] Chang J.-B. , Chen F., Yoon Y.-G., Jung E.E., Babcock H., Kang J.S., Asano S., Suk H.-J., Pak N., Tillberg P.W. et al. Iterative expansion microscopy. Nat. Methods. 2017; 14:593–599. 28417997 10.1038/nmeth.4261 PMC5560071

