# Supplementary material for: Expansion microscopy allows high resolution single cell analysis of epigenetic readers
Source: Nucleic Acids Res. 2022 Jun 18;50(17):e100. doi: 10.1093/nar/gkac521 (PMC9508849; doi:10.1093/nar/gkac521)
Supplement: gkac521_Supplemental_File [file gkac521_supplemental_file.pdf]

Supporting Information

Expansion microscopy allows high resolution single cell analysis of epigenetic readers

Aline Acke[1], Siska Van Belle[2], Boris Louis[1,3], Raffaele Vitale[4], Susana Rocha[1], Thierry Voet[5,7], Zeger Debyser[2] and Johan Hofkens[1,6,7]**

[1] Laboratory for Photochemistry and Spectroscopy, Department of Chemistry, KU Leuven, Leuven, Flanders, Belgium

[2] Laboratory for Molecular Virology and Gene Therapy, Department of Pharmaceutical and Pharmacological Sciences, KU Leuven, Leuven, Flanders, Belgium

[3] Division of Chemical Physics and NanoLund, Lund University, Lund, Sweden

[4] Dynamics, Nanoscopy and Chemometrics (DYNACHEM) Group, U. Lille, CNRS, LASIRE, Laboratoire Avancé de Spectroscopie pour les Interactions, la Réactivité et l'Environnement, Cité Scientifique, F-59000 Lille, France

[5] Department of Human Genetics, KU Leuven, Leuven, Flanders, Belgium

[6] Max Plank Institute for Polymer Research, Ackermannweg 10, Mainz, D-55128, Germany

[7] LISCO, KU Leuven Institute for Single-Cell Omics, Leuven 3000, Belgium

* Shared senior authorship, address correspondence to:

zeger.debyser@kuleuven.be RK-Herestraat 49 – Bus 1023, 3000 Leuven

johan.hofkens@kuleuven.be Celestijnenlaan 200F, 3001 Heverlee (Leuven)

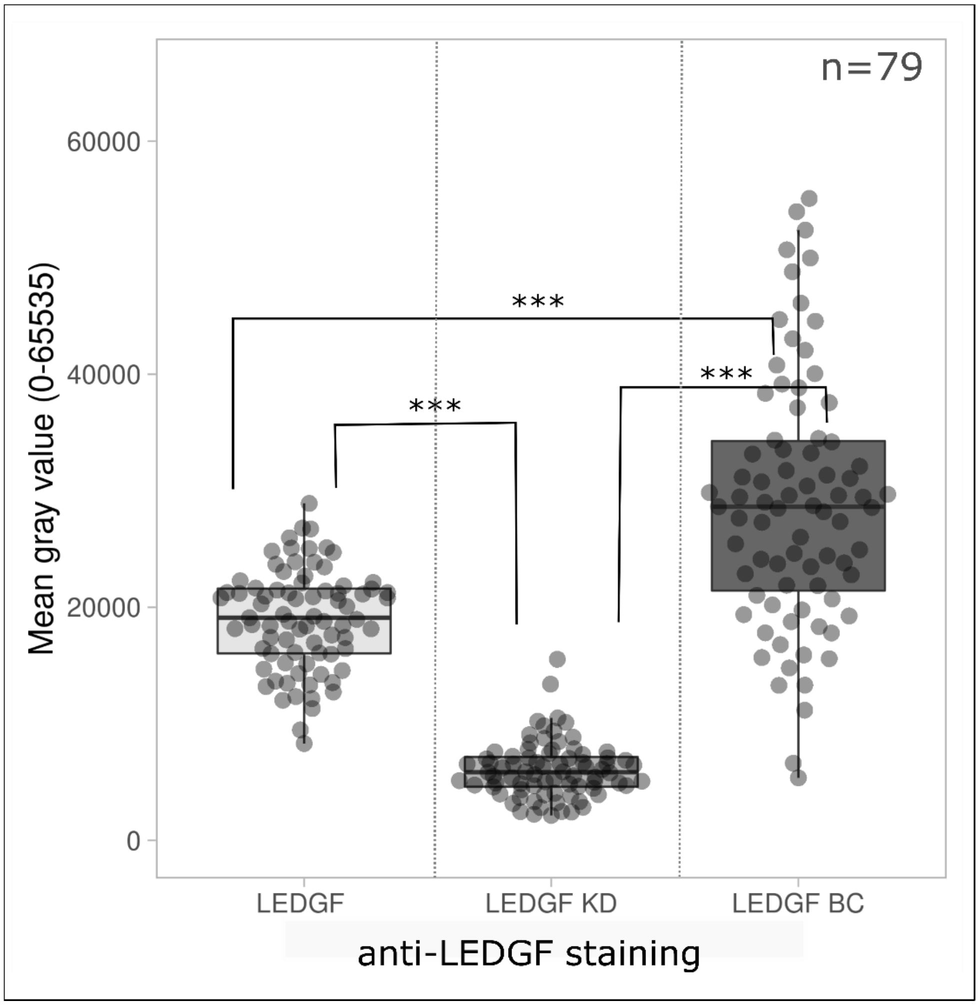

Supplementary Figure 1. Mean intensity values of varying LEDGF/p75 expression levels in HeLaP4 cells via an immunofluorescence assay. Boxplot for cells with WT LEDGF expression (LEDGF), LEDGF/p75 depletion (LEDGF KD) and after LEDGF back complementation (LEDGF BC). Statistical analysis was performed by a One-way ANOVA: ***) p-value < 0.001; Number of cells (n) = 79 with each grey dot representing the mean intensity value in one cell. Standard deviation and mean values can be found in the next table. The primary antibody detects both LEDGF/p75 and LEDGF/p52 (2 µg/ml), the secondary antibody carries an Alexa488 dye (4 µg/ml).

Supplementary Table 1. Measured intensity values for varying LEDGF expression levels in HeLaP4 cells with (n) number of cells, (sd) standard deviation and (sem) standard error of the mean. Differences between groups were assessed by a one-way ANOVA. The values are represented in **SI Fig. 1**.

	Condition	***n***	***mean***	***sd***	***sem***	***median***
1	LEDGF WT	79	18996	4320	489	19105
2	LEDGF BC	79	28961	10886	1233	28636
3	LEDGF/p75 KD	79	6048	2430	275	5844
					**One-way ANOVA**	***P-value***
					LEDGF WT vs LEDGF/p75 KD	*1.76E-52*
					LEDGF WT vs LEDGF BC	*3.18E-12*
					LEDGF/p75 KD vs LEDGF BC	*1.43E-40*

A

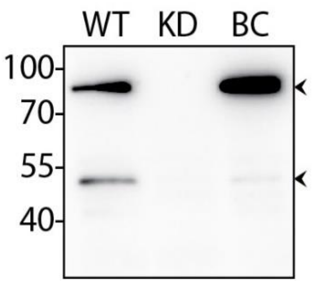

	WT	KD	BC
p75	25965785	0	56764253
p52	14628480	175536	738506
%	100	0.4	142

B

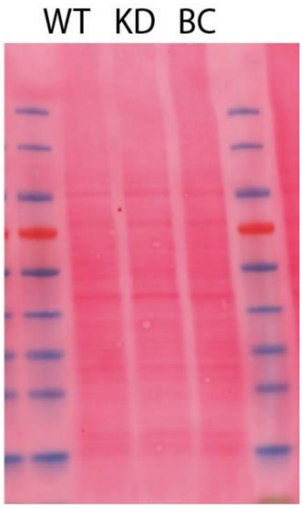

Supplementary Figure 2. Western blot of HeLaP4 cells with different LEDGF/p75 expression levels. A) Analysis of LEDGF expression in cell lysates from wild type HeLaP4 cells (WT), HeLaP4 cells expressing a LEDGF/p75 specific miRNA (KD) and reversed KD by LEDGF/p75 overexpression (BC or back complementation). Arrow heads indicate expected size for LEDGF/p75 (upper arrow head) and LEDGF/p52 (lower arrow head). Expression levels of both p75 and p52 were quantified using ImageJ. Calculated values and the total relative expression compared to WT were listed in the table. **B)** Ponceau staining (right) shows equal loading for all samples.

Supplementary Table 2. Number of LEDGF spots before expansion in HeLaP4 cells with varying LEDGF expression levels with (n) number of cells, (sd) standard deviation and (sem) standard error of the mean. Differences between groups were assessed by a one-way ANOVA. The values are represented in **Fig. 2G**.

	Condition	*n*	*mean*	*sd*	*sem*	*median*
1	LEDGF WT	40	152	63	10	133
2	LEDGF BC	40	117	56	9	109
3	LEDGF/p75 KD	40	126	39	6	119
					**One-way ANOVA**	***P-value***
					LEDGF WT vs LEDGF/p75 KD	*0.03*
					LEDGF WT vs LEDGF BC	*0.01*
					LEDGF/p75 KD vs LEDGF BC	*0.41*

Supplementary Table 3. Number of LEDGF spots after expansion in HeLaP4 cells with varying LEDGF expression levels with (n) number of cells, (sd) standard deviation and (sem) standard error of the mean. Differences between groups were assessed by a one-way ANOVA. The values are represented in **Fig. 2H**.

	Condition	***n***	***mean***	***sd***	***sem***	***median***
1	LEDGF WT	40	1109	265	42	1117
2	LEDGF BC	40	1172	575	91	1098
3	LEDGF/p75 KD	40	737	244	39	744
					**One-way ANOVA**	***P-value***
					LEDGF WT vs LEDGF/p75 KD	*5.80E-09*
					LEDGF WT vs LEDGF BC	*0.530*
					LEDGF/p75 KD vs LEDGF BC	*3.27E-05*

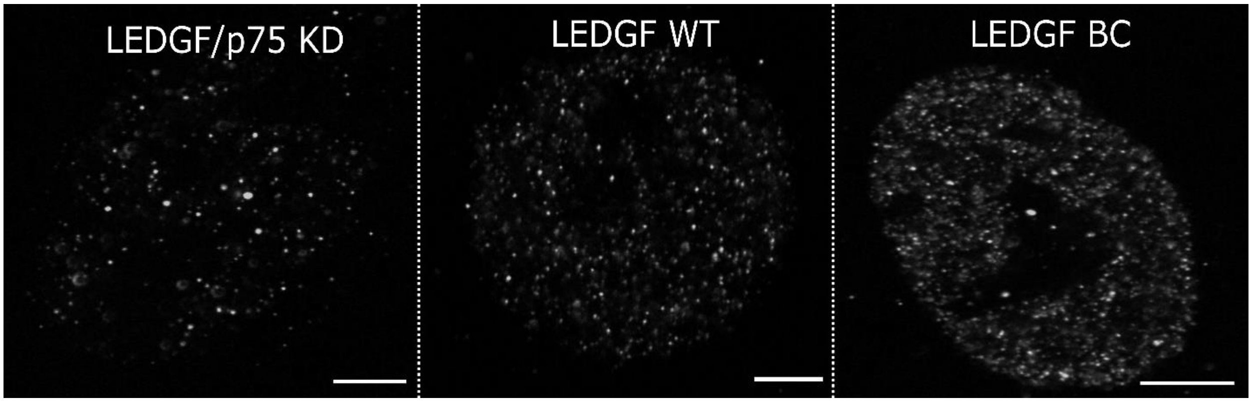

Supplementary Figure 3. Immunofluorescence images of varying LEDGF/p75 expression levels in HeLaP4 cells after expansion. HeLa P4 cells with LEDGF/p75 depletion (LEDGF KD), wild-type expression (LEDGF WT) and after LEDGF back complementation (LEDGF BC). The primary antibody detects both LEDGF/p75 and LEDGF/p52 (2 µg/ml), the secondary antibody carries an Alexa488 dye (4 µg/ml). The presented images are single optical sections. Scale bars: 10 µm.

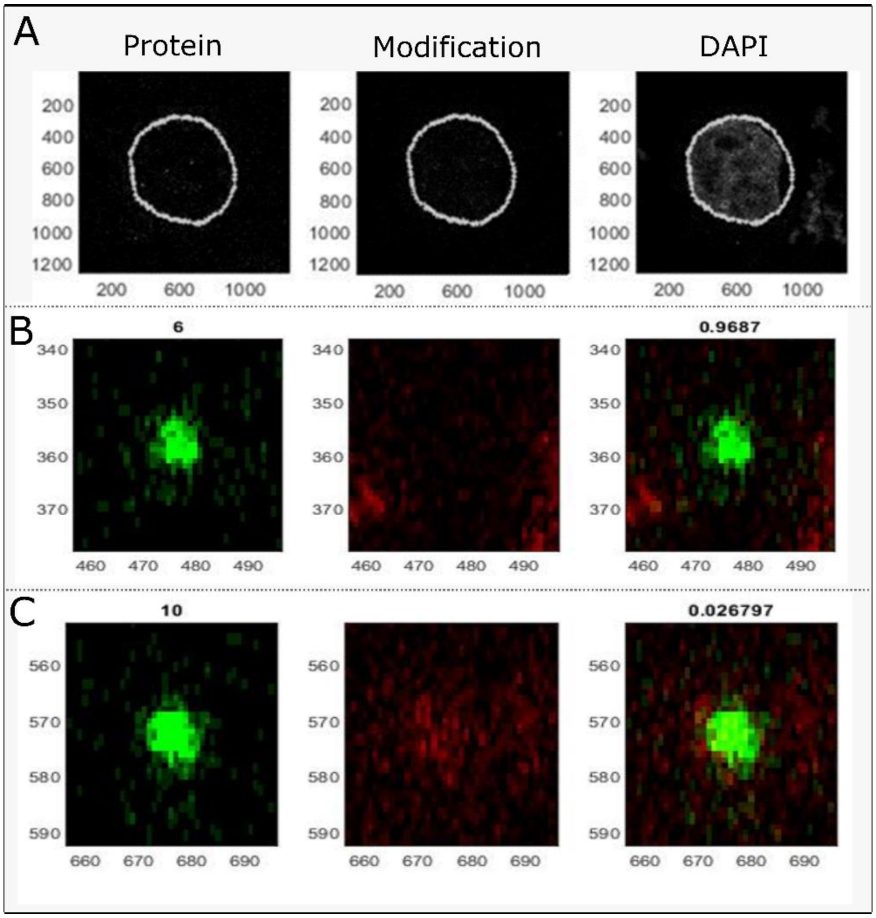

Supplementary Figure 4. Principle of the in-house written MATLAB co-localization routine. **A**) Three fluorescent stainings were used and respective fluorescence signals were measured in three wavelength channels to identify protein locations, epigenetic modifications and to segment the nuclei (DAPI). **B**) A protein spot is detected as a particle (left panel) and its corresponding location is used to detect signal in the epigenetic marker channel (middle panel). The last panel shows a composite image associated to a calculated *p*-value of 0.969. **C**) Same as in **B** but for a distinct protein spot, which corresponds to a stronger epigenetic marker signal and therefore results in a lower *p*-value (0.027). Green channel: primary antibody detecting LEDGF/p75 and p52, secondary antibody with Alexa488 dye; red channel: primary antibody against H3K36me3 modification; secondary antibody with Atto647N dye.

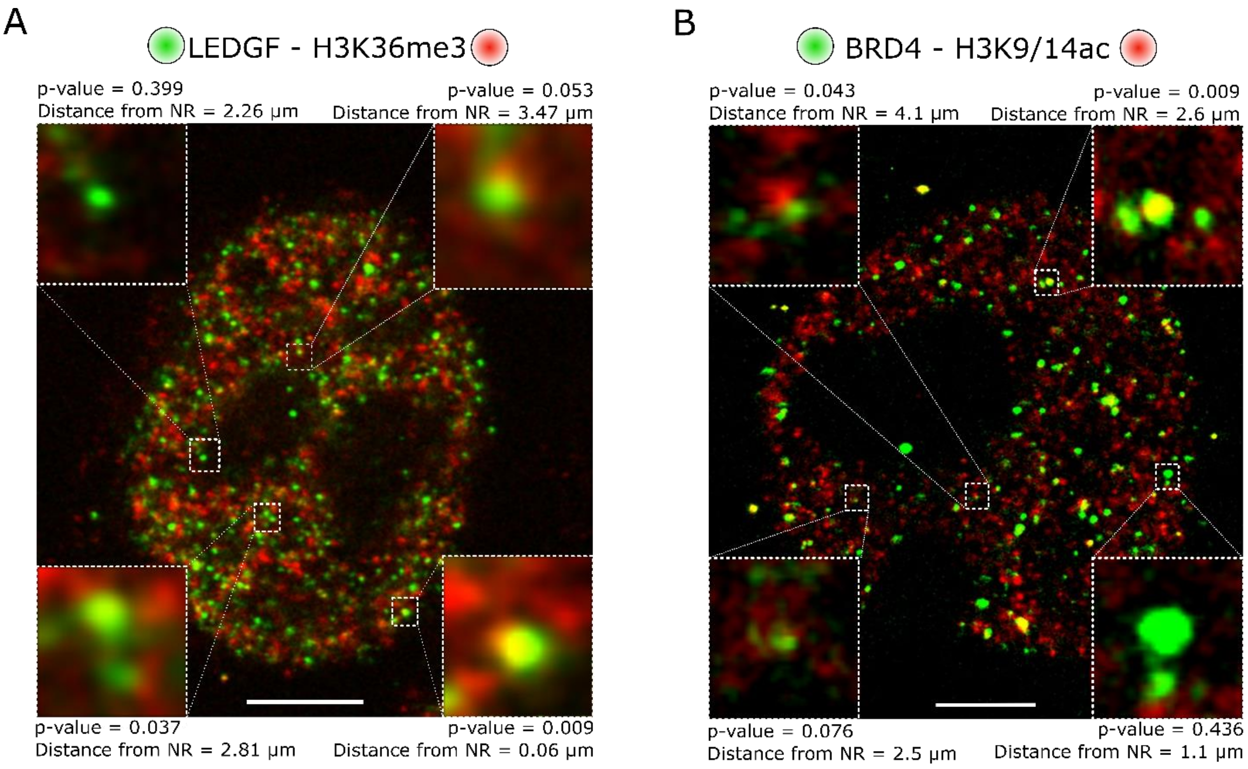

Supplementary Figure 5. Co-localization of an epigenetic reader with a specific euchromatin marker. **A)** Immunostaining of LEDGF (green) and H3K36me (red) with a zoom of four different protein spots depicted in each corner. The upper left and right zoom show no co-localization (*p*-value > 0.05) whereas the bottom zooms do show co-localization with H3K36me3 (*p*-value <0.05). **B**) Immunostaining of BRD4 (green) and H3K9/14ac (red) with a zoom of four different protein spots depicted in each corner. The upper left and right zoom show co-localization (*p*-value < 0.05) whereas the bottom zooms do not show co-localization with H3K9/14ac (*p*-value > 0.05). Each spot also shows the distance from the nuclear rim (NR) in µm after dividing by an average expansion factor of ~3.5. The presented images (A, B) are single optical sections. Scale bars: 10 µm.

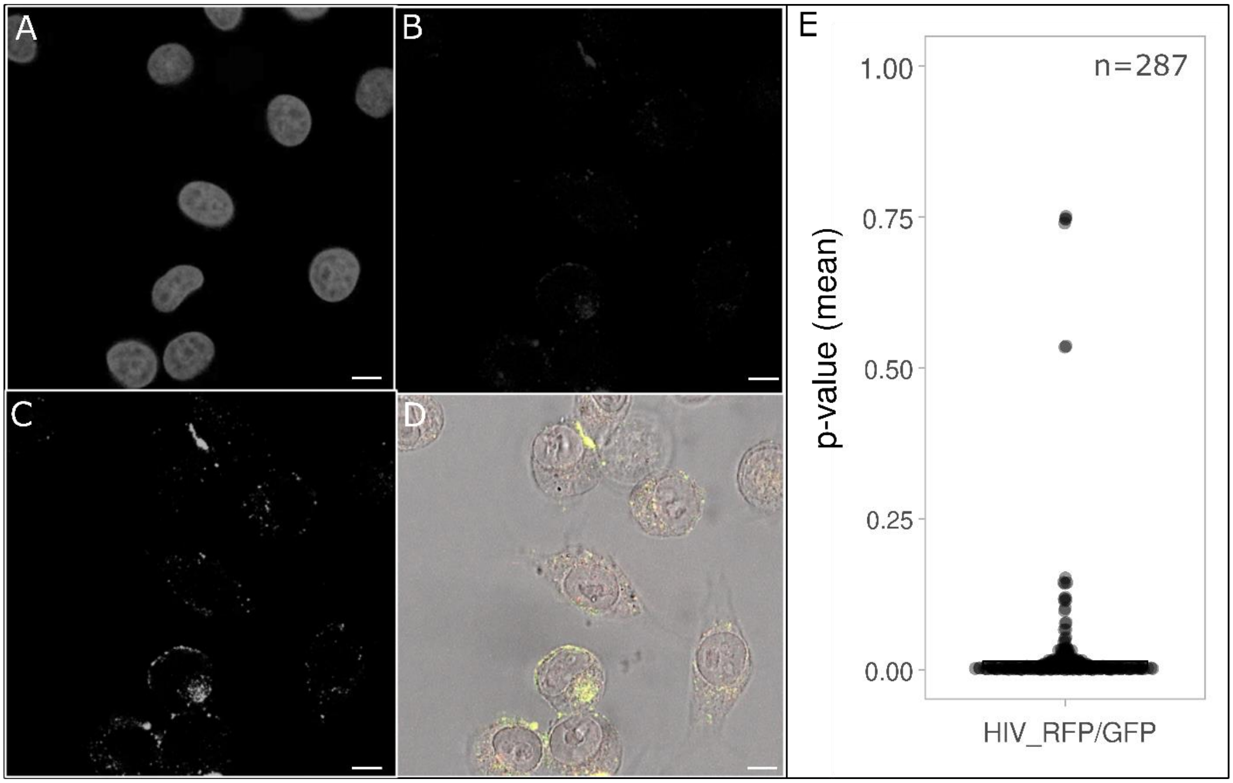

Supplementary Figure 6. Use of dual color HIV-1 particles (Vpr_eGFP/Vpr_mCherry tandem) in HeLaP4 cells fixed 2h after infection as a positive control to validate the analysis pipeline. **A)** DAPI staining to identify the nuclei of the cells. **B**) Expression of mCherry inside the viral particles. **C**) Expression of eGFP inside the viral particles. **D**) Composite bright-field image of the cells with mCherry and eGFP co-localization appearing in yellow. **E**) Box plot of all obtained *p*-values for the detected particles where one grey dot represents one particle; n = 287. The presented images (A-D) are single optical sections. Scale bars: 10 µm (A-D).

Supplementary Table 4. Number of co-localizing spots of LEDGF (p75 and p52) with H3K36me3 when HeLaP4 cells with varying expression levels of LEDGF/p75 are used. (n) denotes number of cells, (sd) standard deviation and (sem) standard error of the mean. Differences between groups were assessed by a one-way ANOVA. The values are represented in **Fig. 4A**.

	Condition	***n***	***mean***	***sd***	***sem***	***median***
1	LEDGF WT	50	124	48	7	123
2	LEDGF BC	50	157	90	13	133
3	LEDGF/p75 KD	50	77	34	5	74

**One-way ANOVA**	***P-value***
LEDGF WT vs LEDGF/p75 KD	*1.35E-07*
LEDGF WT vs LEDGF BC	*0.023*
LEDGF/p75 KD vs LEDGF BC	*4.83E-08*

Supplementary Table 5. Co-localization of the LEDGF (p75 and p52) with specific epigenetic modifications (R). (n) denotes number of cells, (sd) standard deviation and (sem) standard error of the mean. The values are represented in **Fig. 4B**.

	Condition	*n*	*mean (R)*	*sd*	*sem*	*median (R)*
1	H3K27me3	50	6.13	1.12	0.16	6
2	H3K36me2	50	13.87	2.48	0.35	13.72
3	H3K9/14ac	50	9.72	1.16	0.16	9.8
4	H3K36me3	50	12.92	2.35	0.33	13.21

Supplementary text for supplementary Figure 7-10

We reversed the analysis pipeline and measured how many epigenetic marks were occupied by LEDGF/p75 and p52 (**Supplementary Fig. 7**). When the outcome of this analysis for cells with varying LEDGF/p75 expression levels (**Supplementary Fig. 7a, Supplementary Table 6**) was compared to the original one (**Fig. 4A**), there was no significant difference observed in the number of co-localizing spots for both LEDGF BC (148 ± 62 spots; p-value > 0.05) and LEDGF WT cells (113 ± 32 spots; p-value > 0.05) whereas the overall number of co-localizing spots for LEDGF/p75 KD cells appeared to be significantly increased (108 ± 39 spots; p-value < 0.001). The difference in the results (Fig. 4a versus SI Fig. 7a) may be due to the actual binding events but could also be due to the masking of the marker. The immunofluorescence images of H3K36me3 (**Supplementary Fig. 8B**) reveal less defined spots than for LEDGF (**Supplementary Fig. 8A**) hampering the correct detection of the marker in comparison to the epigenetic reader (**Supplementary Fig. 9**). More specifically, the counted number of H3K36me3 spots varies between cells (**Supplementary Fig. 8C and Supplementary Table 7**) with LEDGF WT cells containing 979 ± 265 spots, LEDGF/p75 KD cells 1137 ± 361 spots and LEDGF BC cells 1195 ± 484 spots.

For the marker analysis (**Supplementary Fig. 7B, Supplementary Table 8**) both H3K27me3 (6.29 ± 1.3 R; p-value > 0.05) and H3K9/14ac (9.93 ± 1.22 R; p-value > 0.05) did not show a significant difference in co-localization ratios when compared with earlier results **(Fig. 4B**). The lowest ratio of co-localization was again observed with H3K27me3. For H3K36me2 a significant increase (16.59 ± 3.74 R; p-value < 0.001) was observed in comparison with the analysis displayed in Figure 4b and with the ratio obtained for H3K36me3 (13.59 ± 3.45 R). To determine if this variation between H3K36me3 and H3K36me2 is given by a difference in specificity and/or affinity of the used antibodies, a western blot was performed (**Supplementary Fig. 10**). Detection of recombinant nucleosomes with both modifications, H3K36me2 and H3K36me3 (**Supplementary Fig. 10A**), showed no non-specific binding of the monoclonal H3K36me2 antibody. However, there was some detection of H3K36me2 when the polyclonal H3K36me3 antibody was used. To determine the affinity of both antibodies, two dilutions (1/1000 and 1/2000) of the stock concentrations were used in a western blot (**Supplementary Fig. 10B**). By quantifying the density of the acquired bands and taking into account the difference in stock concentrations, a ~20-fold drop in density was estimated for the H3K36me2 antibody when compared to the H3K36me3 antibody, indicating a lower affinity of the former. These results indicate how both antibody specificity and affinity play an important role in the correct detection of the marker.

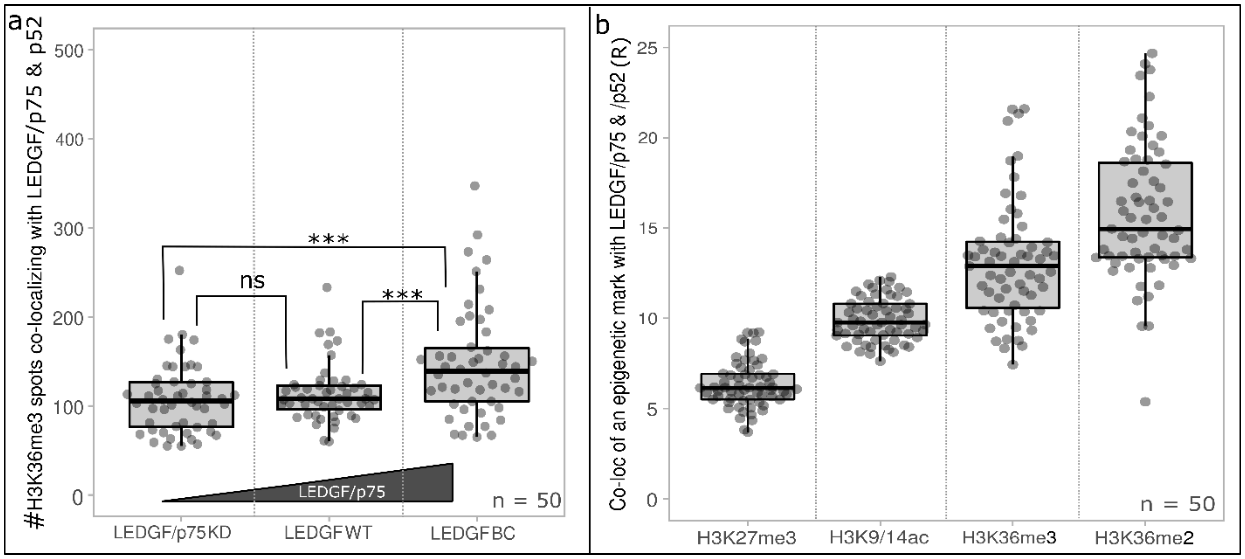

Supplementary Figure 7. Reversed analysis for co-localization of epigenetic modifications with LEDGF/p75 and p52. A) The number of H3K36me3 spots co-localizing with LEDGF was measured in HeLaP4 cells (LEDGF WT) and in LEDGF/p75 KD or BC cells. **B)** Co-localization of H3K27me3 – H3K9/14ac – H3K36me3 and H3K36me2 marks with LEDGF. Statistical analysis was performed by a One-way ANOVA: ns) non-significant; ***) *p*-value < 0.001; Number of cells (n) = 50 with each grey dot representing one cell. Co-localization analysis was performed on Z-stacks of expanded cells consisting of 9-11 Z-slices with a Z-step size of 0.2 µm.

Supplementary Table 6. Number of H3K36me3 spots co-localizing with LEDGF (p75 and p52) when HeLaP4 cells with varying expression levels of LEDGF/p75 are used. (n) denotes number of cells, (sd) standard deviation and (sem) standard error of the mean. Differences between groups were assessed by a one-way ANOVA. The values are represented in **SI Fig. 7A**.

	Condition	*n*	*mean*	*sd*	*sem*	*median*
1	LEDGF WT	50	114	32	5	108
2	LEDGF BC	50	148	62	9	139
3	LEDGF/p75 KD	50	108	39	6	106

**One-way ANOVA**	***P-value***
LEDGF WT vs LEDGF/p75 KD	*0.411*
LEDGF WT vs LEDGF BC	*0.0009*
LEDGF/p75 KD vs LEDGF BC	*0.0002*

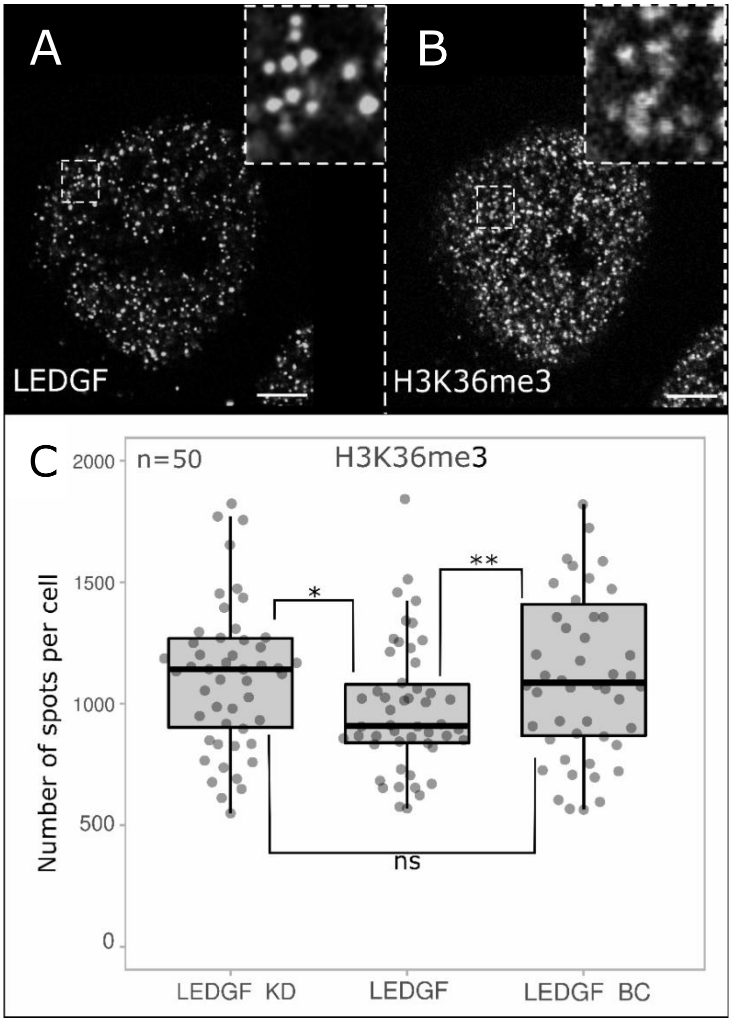

Supplementary Figure 8. Counting of distinct epigenetic marks in HeLaP4 cells with varying LEDGF expression levels A-B) Typical post-expansion image of LEDGF (**A**) and H3K36me3 (**B**); the upper right corner depicts a zoom of the boxed area. **C**) Number of spots counted for H3K36me3 in three different cell lines: LEDGF (WT) – LEDGF/p75 KD – LEDGF BC. Statistical analysis was performed by a One-way ANOVA: ns) non-significant; *) p-value < 0.05; **) p-value < 0.01; number of cells (n) = 50. The presented images (a, b) are single optical sections. Scale bars: 10 µm (A-C). LEDGF (p75 and p52) primary antibodies (2 µg/ml) are stained with GAM Alexa488 secondary antibodies and the H3K36me3 antibody (1 µg/ml) with GAR Atto647N.

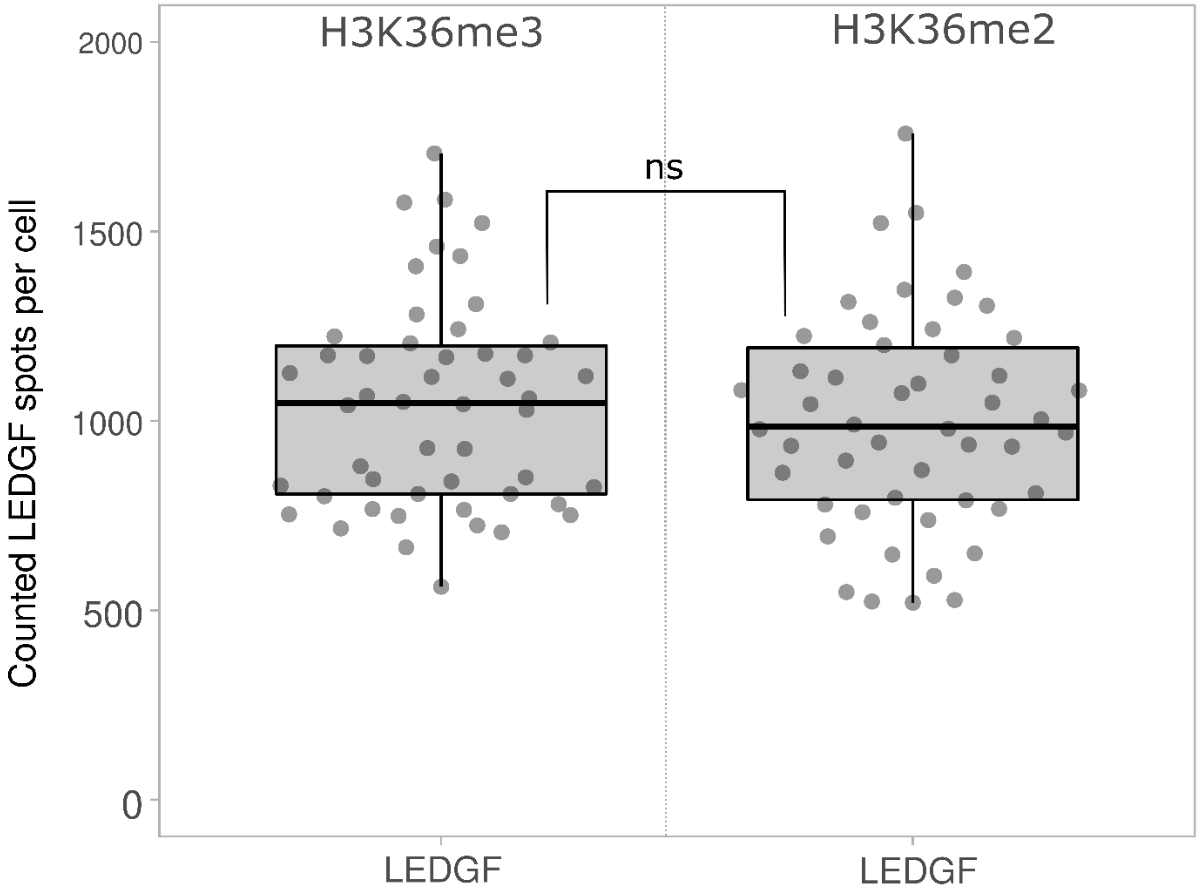

Supplementary Figure 9. Number of LEDGF WT protein spots in HeLaP4 cells while detecting different epigenetic modifications. Statistical analysis was performed by a One-way ANOVA: ns) non-significant; *p*-value > 0.05; number of cells (n) = 50. Details on used antibodies and dilutions can be found in **SI Table 11**.

Supplementary Table 7. Rows 1-3 contain the number of H3K36me3 spots in HeLaP4 cells with varying LEDGF expression levels. (n) denotes number of cells, (sd) standard deviation and (sem) standard error of the mean. Differences between groups were assessed by a one-way ANOVA. The values are represented in **SI Fig. 8C**.

	Condition	*n*	*mean*	*sd*	*sem*	*median*
1	H3K36me3 (LEDGF BC)	50	1195	484	69	1087
2	H3K36me3 (LEDGF/p75 KD)	50	1137	361	52	1142
3	H3K36me3 (LEDGF WT)	50	979	265	38	909
					**One-way ANOVA**	***P-value***
					LEDGF/p75 KD vs LEDGF WT	*0.014*
					LEDGF WT vs LEDGF BC	*0.007*
					LEDGF/p75 KD vs LEDGF BC	*0.496*

Supplementary Table 8. Co-localization of a specific epigenetic modification with LEDGF (R). (n) denotes number of cells, (sd) standard deviation and (sem) standard error of the mean. The values are represented in **SI Fig. 7B**.

	Condition	*n*	*mean (R)*	*sd*	*sem*	*median (R)*
1	H3K27me3	50	6.29	1.3	0.19	6.14
2	H3K36me2	50	16.59	3.74	0.53	16.44
3	H3K9/14ac	50	9.93	1.22	0.17	9.71
4	H3K36me3	50	13.59	3.45	0.49	13.34

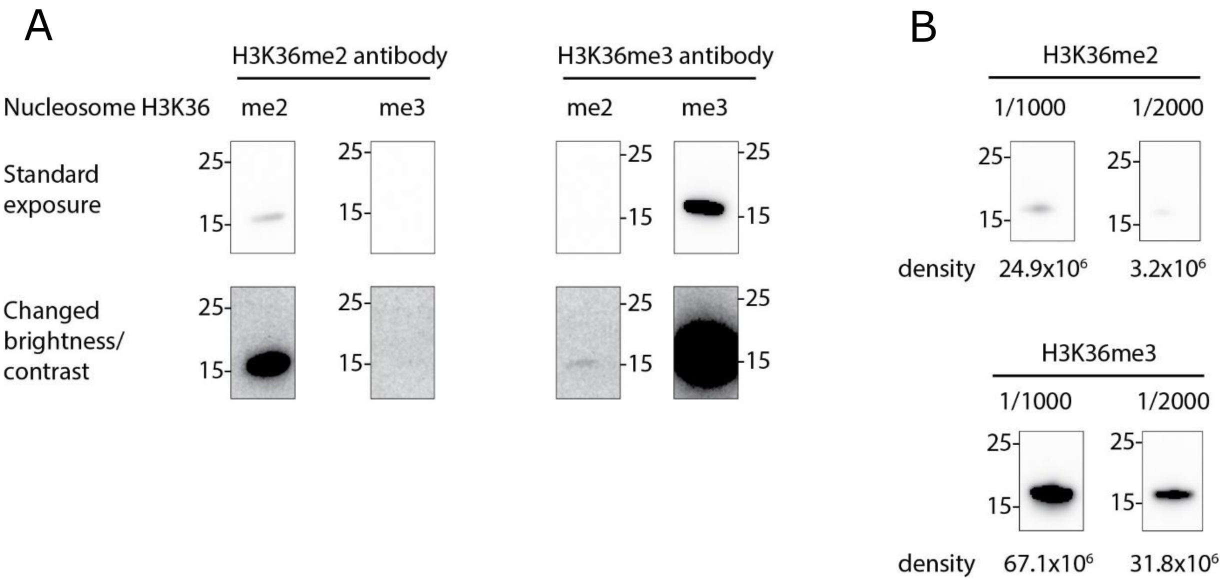

Supplementary Figure 10. Western blot of H3K36me3 and H3K36me2 antibody to determine affinity and specificity. A) Detection of recombinant nucleosomes with specific H3K36 di- or trimethyl modifications (me2, me3) by specific H3K36 di- (left) or trimethyl (right) recognizing antibodies. Bottom: modified brightness and contrast to visualize weaker bands. B) Top: Detection of nucleosomes with H3K36me2 modification by two concentration of H3K36me2 antibody. Dilution from the stock concentration (0.9 mg/ml). Bottom: Similar setting for H3K36me3 nucleosome and antibody. Stock concentration 0.4 mg/ml. Bands were analyzed using ImageJ: the resulting density value is indicated under each blot.

Supplementary text for supplementary Figure 11 and 12

The clear band (**Supplementary Fig. 11B**) visible in LEDGF WT HEK239T cells around 75 kDa represents LEDGF/p75 whereas a faint band around ~50 kDa represents LEDGF/p52. No band is detected for LEDGF/p75 in the knock out cells and also p52 appeared only as a weak band. These results correspond with those of intensity-based immunofluorescence analysis (**Supplementary Fig. 12A**), displaying an 80% decrease in intensity in LEDGF/p75 knock out cells when a primary antibody that recognizes both isoforms is used. We attributed the remaining 20% to detection of the p52 isoform that is still expressed. In the attempt of avoiding p52 detection, an antibody specific for LEDGF/p75 was used as well (**Supplementary Fig. 11A**). Although no protein bands of either 75 kDa or 50 kDa were present in the LEDGF/75 knock out cells, aspecific bands of 20 kDa and higher were detected. This aspecific staining may explain the residual staining in the immunofluorescence analysis (**Supplementary Fig. 12B**), with 14% intensity detected in LEDGF/p75 knock out cells when compared to WT cells.

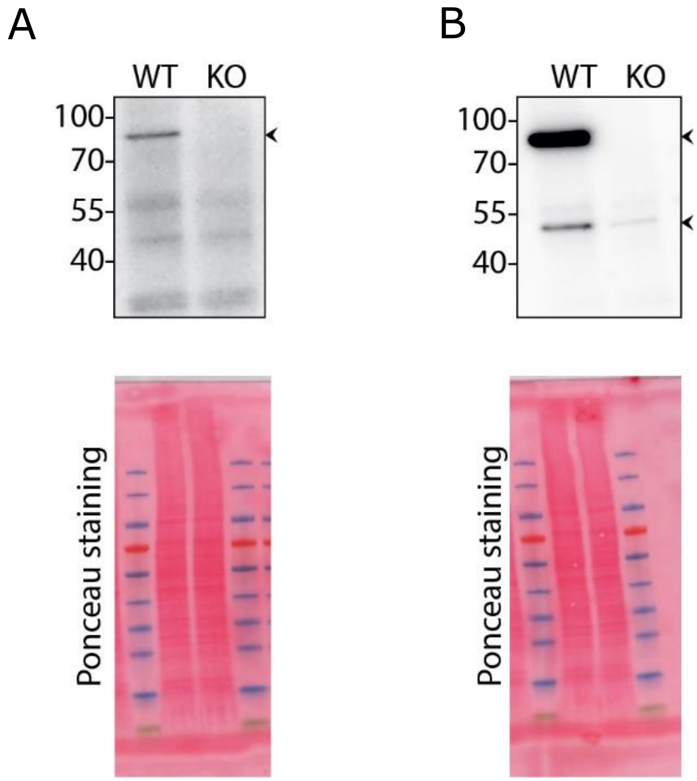

Supplementary Figure 11. Western blot to test specificity of two different LEDGF primary antibodies in HEK293T wild type cells and HEK239T LEDGF/p75 knock out cells. Analysis of LEDGF/p75 expression in cell lysates from wild type HEK293T cells (WT) or LEDGF/p75 specific KO (KO) cells. Detection with either a LEDGF/p75 specific antibody from Bethyl Companies (A) or LEDGF antibody from BD (B). Arrow heads indicate expected size. Bottom: Ponceau staining to indicate equal loading. 20 µg of HEK293T, primary antibody at 1:2000 dilution and secondary at 1:10000.

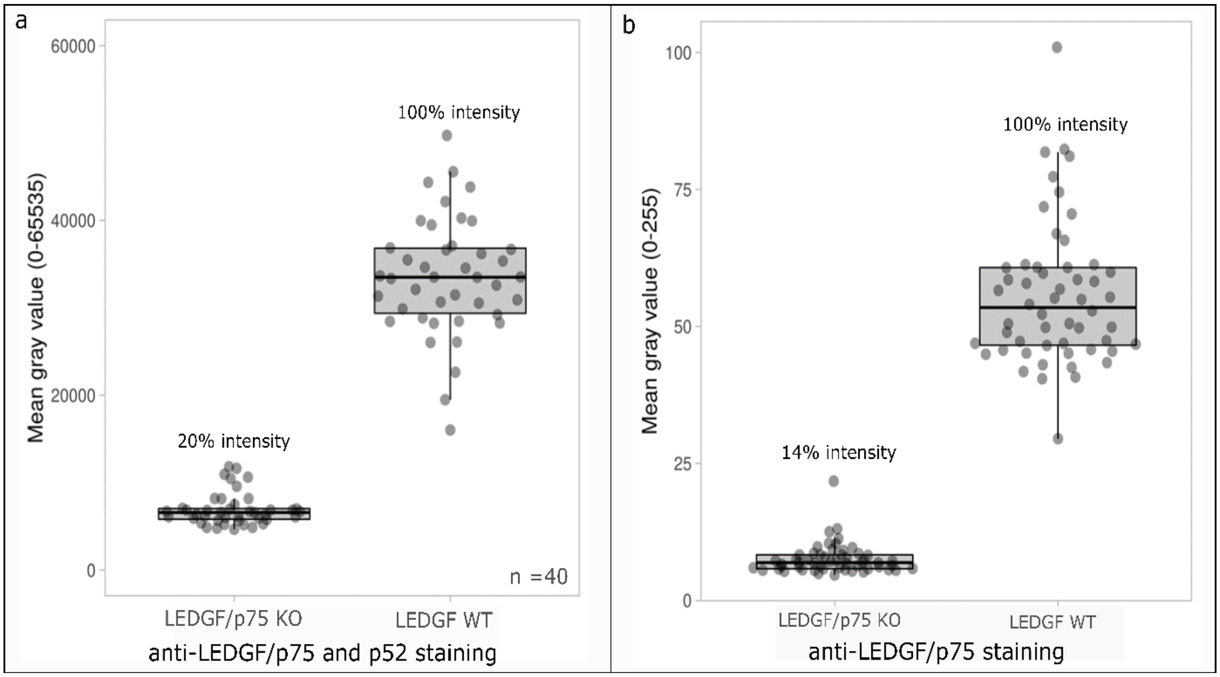

Supplementary Figure 12. Mean intensity values of varying LEDGF expression levels in HEK293T cells via immunofluorescence. A) Detection of both LEDGF/p75 and LEDGF/52 in LEDGF/p75 knock out cells (KO) and cells with WT LEDGF expression. **B)** Detection of LEDGF/p75 in LEDGF/p75 KO cells and LEDGF WT cells. Number of cells (n) = 40 with each grey dot representing one cell. The BD LEDGF antibody was used to detect LEDGF (p75 and p52) in (a) at 2 µg/ml whereas in (b) the Bethyl antibody (0.2 µg/ml) detected only LEDGF/p75 and not p52.

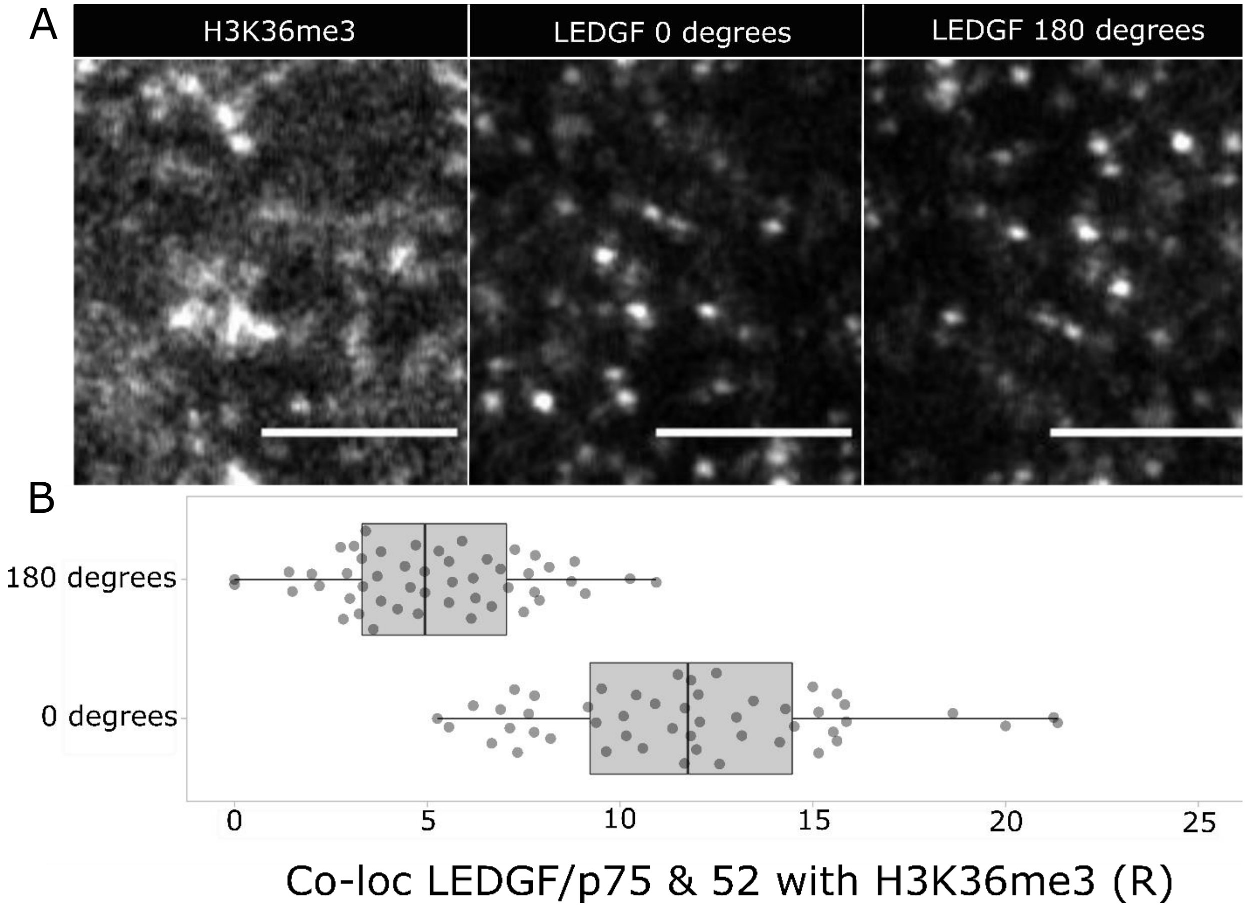

Supplementary Figure 13. Rotation of LEDGF detection channel to calculate random co-localization. A) The LEDGF channel is rotated 180 degrees and matched again with the H3K36m3e channel to calculate the amount random co-localization; Scale bars: 5 µm. **B**) Co-localization of LEDGF (p75 & p52) with H3K36me3. Analysis in the LEDGF channel is done at the original position (0 degrees) and after a rotation of 180 degrees from its original position. Co-localization ratios are plotted in a box plot with each grey dot representing one cell; number of cells (n) = 50. Co-localization analysis was performed on Z-stacks of expanded cells consisting of 9-11 Z-slices with a Z-step size of 0.2 µm.

Supplementary Table 9. Co-localization of BRD4 with specific epigenetic modifications (R). (n) denotes number of cells, (sd) standard deviation and (sem) standard error of the mean. The values are represented in **Fig. 5a**.

	Condition	*n*	*mean(R)*	*sd*	*sem*	*median(R)*
1	H3K27me3	50	11.67	3.38	0.48	10.63
2	H3K36me3	50	13.42	2.32	0.33	13.58
3	H3K9/14ac	50	14.75	2.96	0.42	14.54
4	H3K9me3	50	9.27	3.09	0.44	8.92

Supplementary Table 10. Co-localization of the BRD4 with H3K9/14ac (R) in the presence of varying concentrations of JQ1 (0 nM – 500 nM). (n) denotes number of cells, (sd) standard deviation and (sem) standard error of the mean. Differences between groups were assessed by a one-way ANOVA. The values are represented in **Fig. 5b**.

	Condition	*n*	*mean(R)*	*sd*	*sem*	*median(R)*
1	H3K9/14ac	50	14.75	2.96	0.42	14.54
2	JQ1 (0 nM)	50	15.88	2.89	0.41	15.45
3	JQ1 (25 nM)	50	14.87	3.26	0.46	15.21
4	JQ1 (50 nM)	50	14.1	2.74	0.39	13.57
5	JQ1 (125 nM)	50	12.58	2.95	0.42	12.59
6	JQ1 (250 nM)	50	12.03	2.68	0.38	11.95
7	JQ1 (500 nM)	50	10.62	2.41	0.34	10.5
					**One-way ANOVA**	***P-value***
					H3K9/14ac vs JQ1 (0nM)	*0.056*
					JQ1 (0nM) vs JQ1 (25nM)	*0.103*
					JQ1 (25nM) vs JQ1 (50nM)	*0.204*
					JQ1 (50nM) vs JQ1 (125nM)	*0.009*
					JQ1 (125nM) vs JQ1 (250nM)	*0.333*
					JQ1 (250nM) vs JQ1 (500nM)	*0.007*
					H3K9/14ac vs JQ1 (25nM)	*0.852*
					H3K9/14ac vs JQ1 (50nM)	*0.255*
					H3K9/14ac vs JQ1 (125nM)	*0.0004*

Supplementary text for supplementary Figure 14

We ran an additional analysis to determine whether the epigenetic reader BRD4 mostly resides in regions where chromatin is present. Based on a DNA staining with DAPI, we determined nuclear regions without chromatin. Next, these DAPI data were converted to a binary image (DAPI mask) in FIJI and used in our co-localization analysis with BRD4 to obtain the level of BRD4 foci residing on chromatin (**Supplementary Figure 14**). This analysis revealed that 92.2 ± 3.74 % of the detected BRD4 foci were found in regions with chromatin present in a total of 50 cells (n = 50). This confirms that our application of the method does not differ from what it is intended for since a high numbers of readers is bound to chromatin.

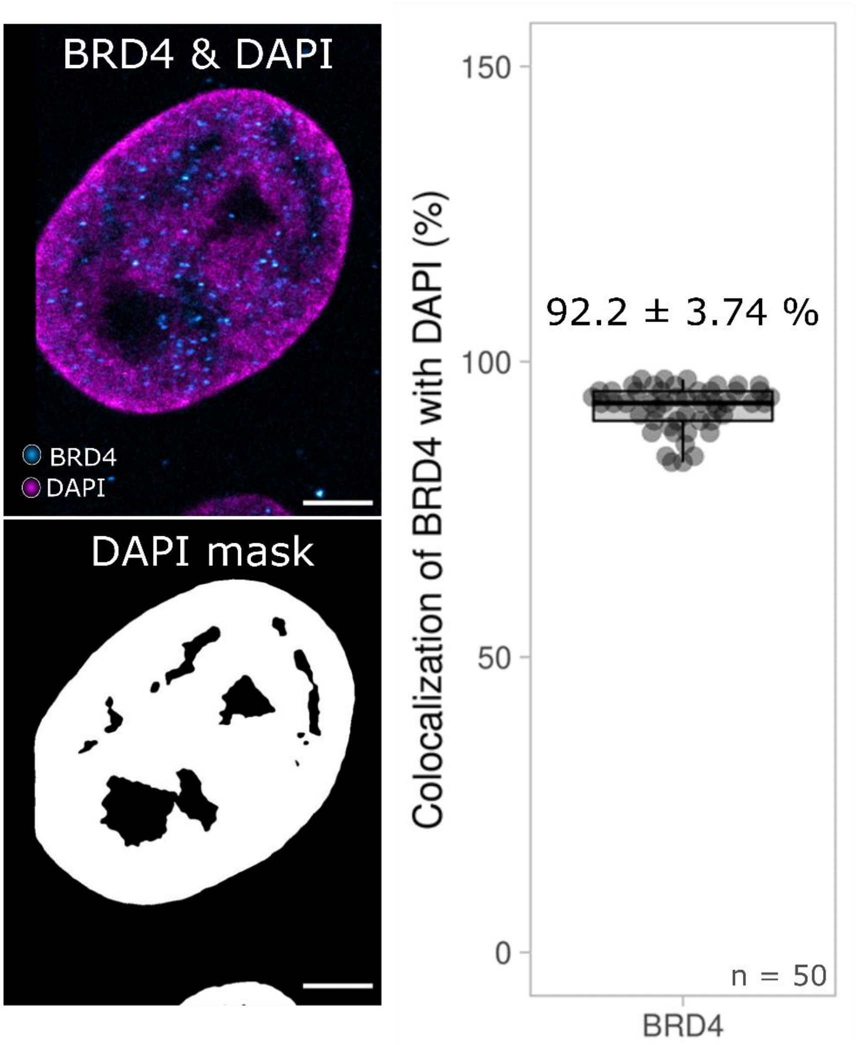

Supplementary Figure 14. Co-localization between DAPI staining and BRD4 immunostained proteins. The top dual-color image displays DNA stained with DAPI (magenta) and BRD4 is immunostained with GAM Alexa488 (cyan). The bottom black and white image is a binary image of the DAPI staining (mask). Co-localization percentages are plotted in a box plot with each grey dot representing one cell; number of cells (n) = 50. The co-localization analysis was performed on Z-stacks of expanded cells consisting of 9-11 Z-slices with a Z-step size of 0.2 µm. Scale bars: 10 µm.

Supplementary text for supplementary Figure 15-16

For BRD4, many studies on phase separation and focus formation have emerged. However we would like to emphasize that we do not merely look at condensates but are also able to detect the free population of BRD4 even with limited resolution. In addition, this research is not aimed at the investigation of phase separation and focus formation but simply demonstrates interactions of different epigenetic readers with their epigenetic landscape. Nevertheless, we investigated the possible detection of BRD4 condensates. This was achieved by increasing the intensity threshold ~4x to detect spots as foci, since a higher number of labelled proteins that cluster together will result in a higher intensity. Indeed, when the intensity threshold (IT) was increased from 70 to 300, less spots were counted, 418 ± 114 foci vs 162 ± 49 foci, respectively when a total of 50 cells (n = 50) is analyzed (**SI Fig.15**). Furthermore, when the assumption is made that chromatin associates with condensates rich in epigenetic readers this would imply that the amount of co-localization with an epigenetic marker, which is in essence chromatin, should increase when looking specifically at condensates. Co-localization with H3K9/14ac indeed increased significantly from 14.75 ± 2.96 R for IT 70, to 17.76 ± 5.12 R for IT 300 in a total of 50 cells (p-value = 0.0005) (**SI Fig.16)**. This may indicate that we are looking more specifically to BRD4 condensates in this case.

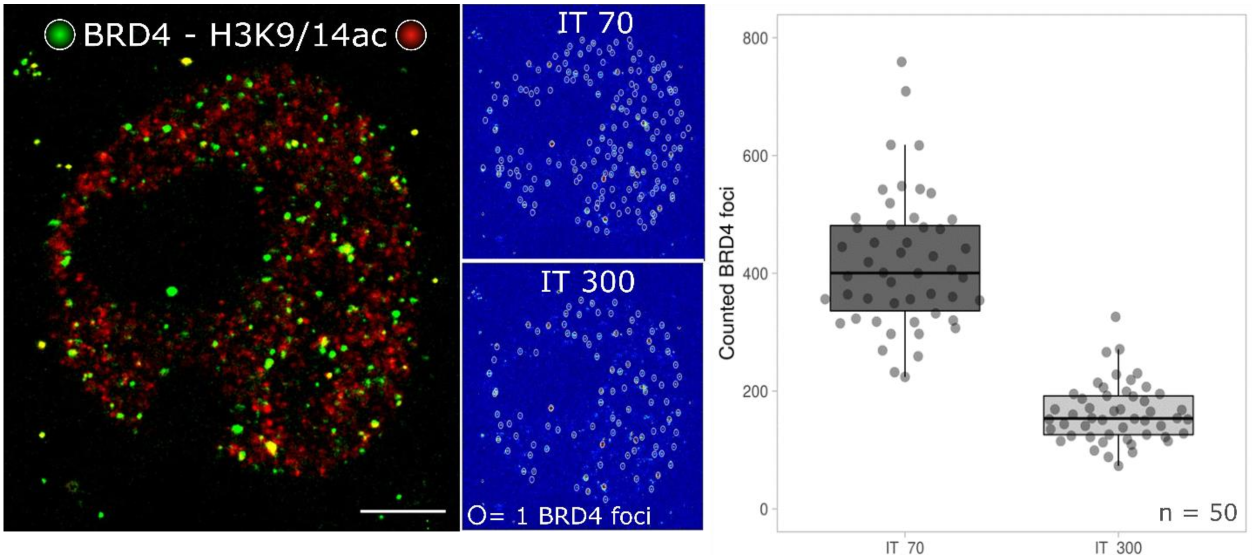

Supplementary Figure 15. Increasing intensity threshold (IT) for BRD4 condensate detection. The left panel displays a dual-color image of immunostained BRD4 with GAM Alexa488 (green) and H3K9/14ac with GAR Atto647N (red). The two middle panels show which BRD4 spots are counted as foci (white circles) with on top the original IT (70) and in the bottom the increased IT (300). In the right panel, counted BRD4 foci are plotted in a box plot with each grey dot representing one cell; number of cells (n) = 50. The analysis was performed on Z-stacks of expanded cells consisting of 9-11 Z-slices with a Z-step size of 0.2 µm. Scale bar: 10 µm.

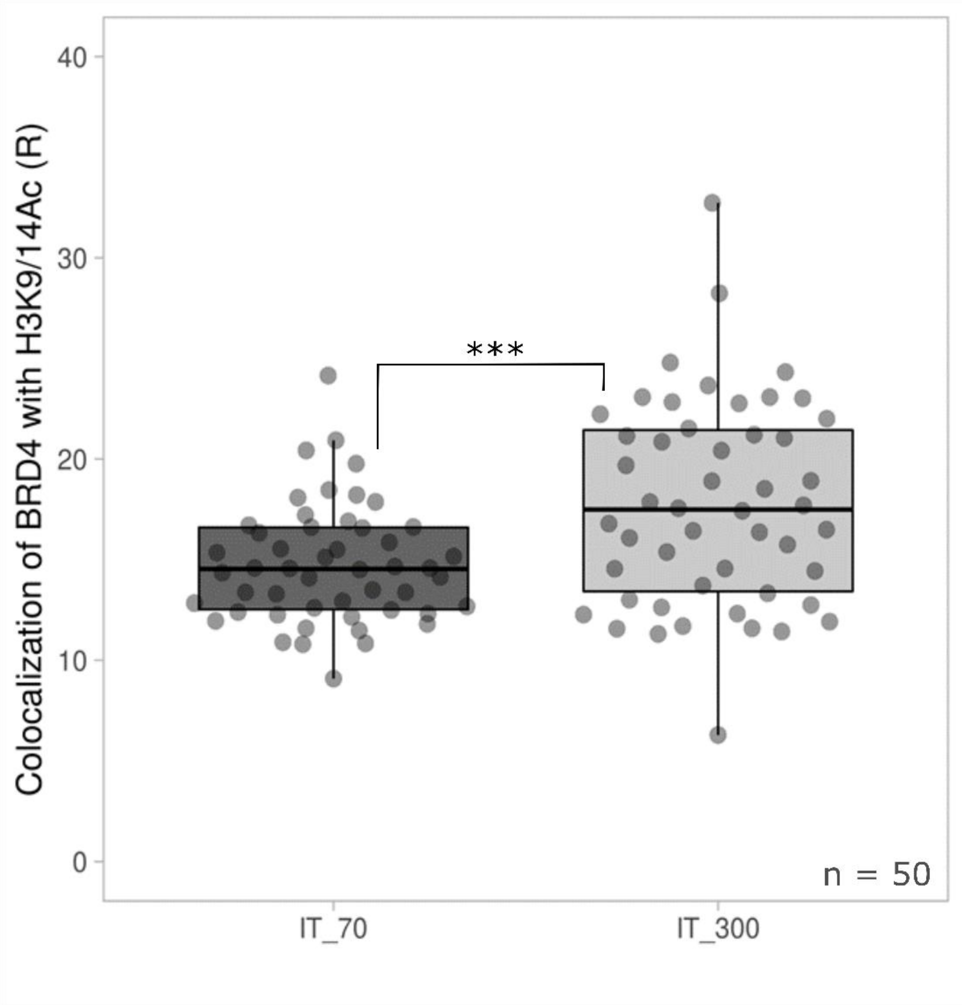

Supplementary Figure 16. Co-localization of BRD4 with H3K9/14ac for two different intensity thresholds (70 and 300). Statistical analysis was performed by a One-way ANOVA: ***) p-value < 0.001; number of cells (n) = 50. Details on used antibodies and dilutions can be found in **SI Table 11**. Co-localization analysis was performed on Z-stacks of expanded cells consisting of 9-11 Z-slices with a Z-step size of 0.2 µm.

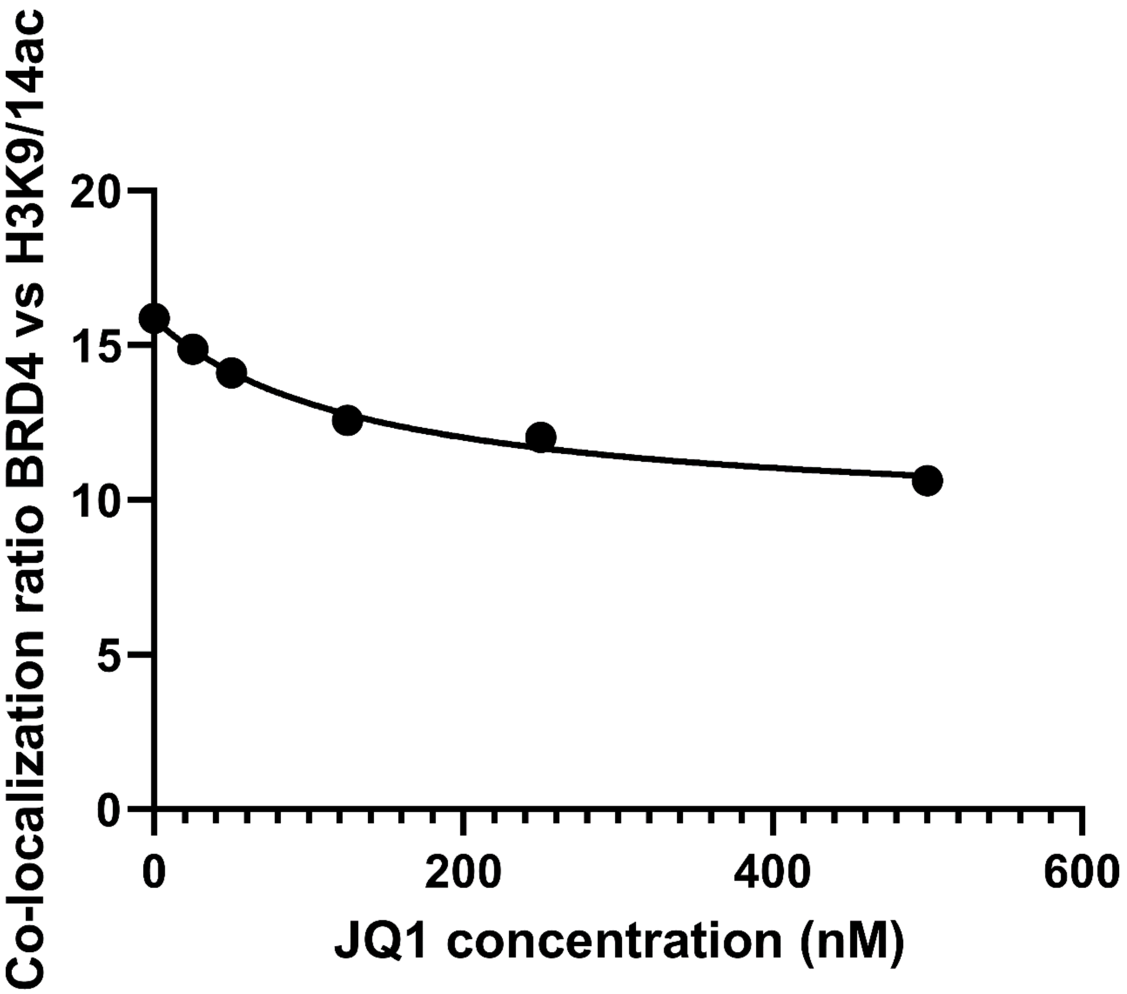

Supplementary Figure 17. An IC_{50} for JQ1 could be calculated based on a non-linear dose-response curve. JQ1 data (0 nM – 500 nM) were used to create a dose-response curve and calculate an IC_{50} of 137 nM (95% confidence interval: 61.48 nM – 395.1 nM) for BRD4 co-localization with H3K9/14ac. Analysis was done in Prism through a non-linear regression curve with standard slope (three parameters).

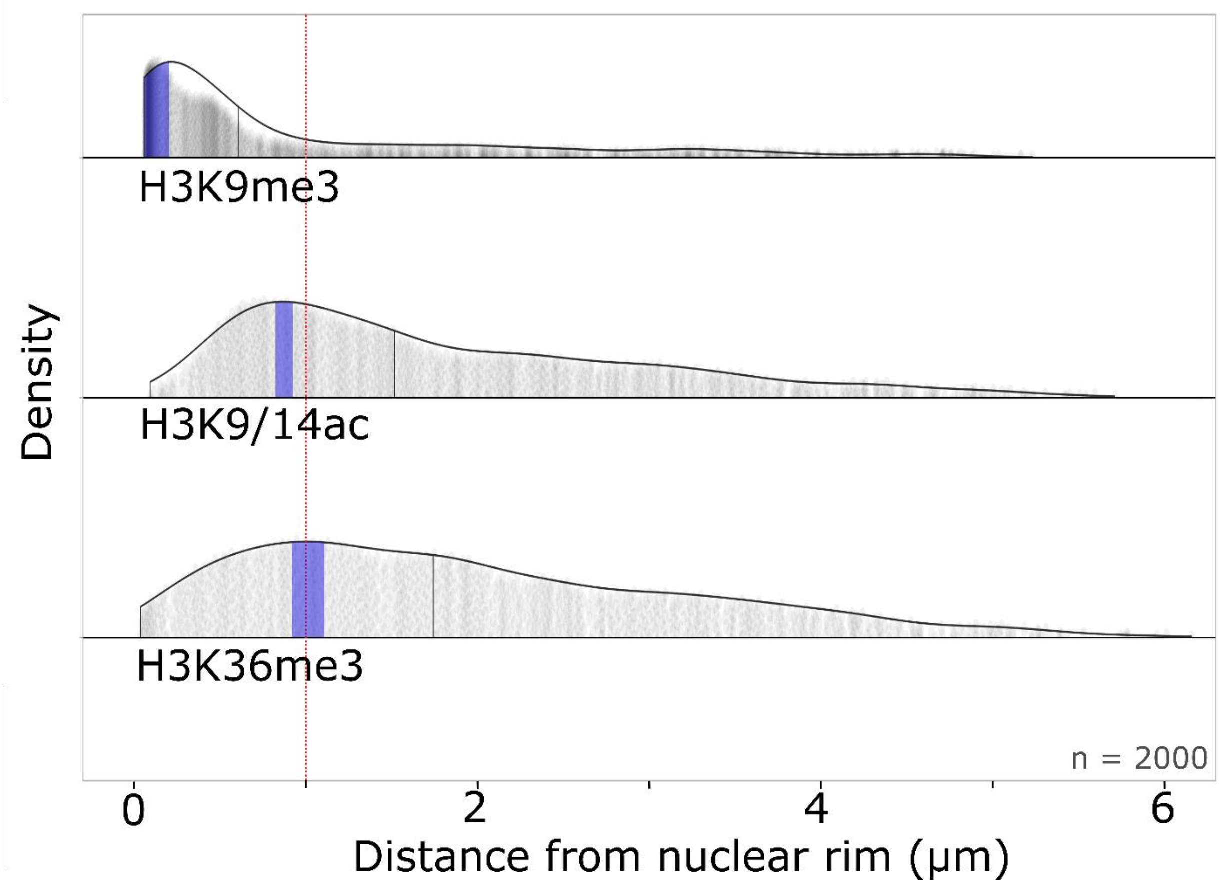

Supplementary Fig. 18. Spatial organization of histone marks in HeLaP4 cells. Density plots for the distance (µm) of H3K9me3, H3K9/14ac and H3K36me3 to the nuclear rim. The median distance is presented as a thin black line inside the density plot: H3K9me3 = 0.45 µm; H3K9/14ac = 1.39 µm; H3K36me3 = 1.71 µm. The region with the highest density is colored blue for each plot: H3K9/14ac ~ 0.1 µm; H3K9/14ac ~ 0.8 µm; H3K36me3 ~ 1.0 µm. The red dotted line inside the density plot represents a distance of 1.0 µm. The average radius of a HeLaP4 nucleus is 6.00 ± 0.69 µm. Number of analyzed spots (n) = 2000 with each grey dot representing one marker spot. The spatial analysis was performed on Z-stacks of expanded cells consisting of 9-11 Z-slices with a Z-step size of 0.2 µm.

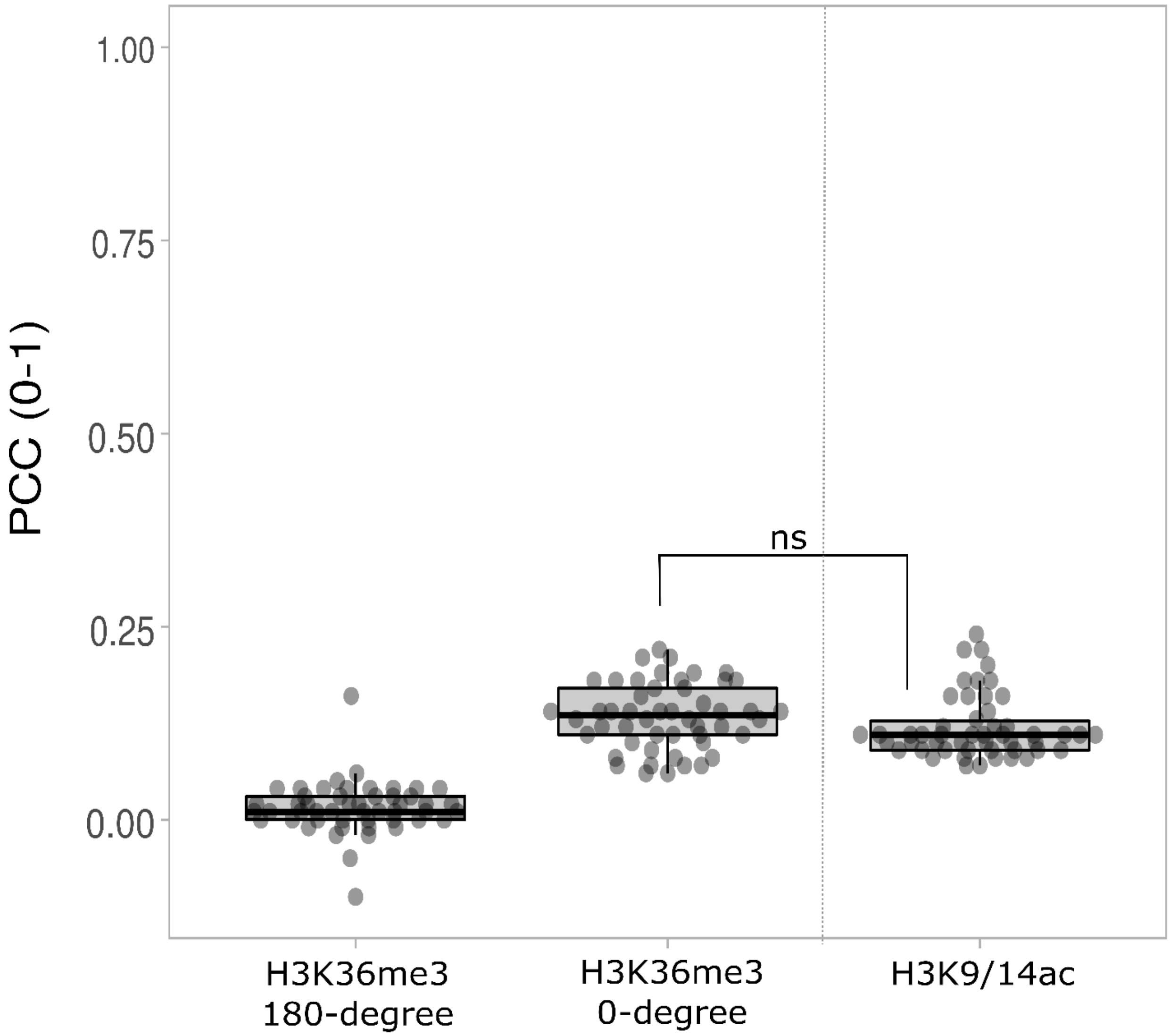

Supplementary Figure 19. Pearson's correlation coefficient (PCC) for co-localization of LEDGF with H3K9/14ac or H3K36me3 (0-degree rotation and 180-degree rotation) in HeLaP4 cells. To determine possible correlations between a marker and LEDGF (p75 and p52), a PCC analysis was performed. Differences between groups were assessed by a one-way ANOVA: ns) non-significant; *p*-value > 0.05 (= 0.093); number of counted cells (n) = 50. Co-localization analysis was performed on Z-stacks of expanded cells consisting of 9-11 Z-slices with a Z-step size of 0.2 µm.

Supplementary Table 11. Materials and compounds used.

NAME	*CONCENTRATION*	*SUPPLIER*
**CELL CULTURE**		
Dulbecco's Modified Eagle Medium (DMEM)	/	Gibco (31053-028)
Fetal Bovine Serum (FBS)	10% (v/v)	Sigma-Aldrich (F7524)
Gentamicin	50 µg/mL	Gibco (15750060)
Geneticin	500 µg/mL	Sigma-Aldrich (A1720)
Glutamax	5%	Gibco (35050-038)
10x DPBS	1x	Gibco (14080051)
0.5% Trypsin-EDTA (10x)	1x	Gibco (15400-054)
T25 flasks	/	VWR (10062-872)
Zeocin	100 µg/mL	ThermoFisher (R25001)
Blasticidin	5 µg/mL	PanReac Applichemi (A3784,0010)
**IMMUNOSTAINING/ANCHORING**		
29 mm glass-bottom dishes	/	InVitro Scientific (D29-14-1.5-N)
Paraformaldehyde (PFA)	4%	ThermoScientific (28908)
Triton X-100	0.2%	J & K (993361)

Bovine Serum Albumin (BSA)	2%	Sigma-Aldrich (A8412)
Tween-20	0.2%	Sigma-Aldrich (P7949)
Goat serum	5%	Sigma-Aldrich (G9023)
DAPI	1 µg/mL	Invitrogen (D1306)
Acryloyl-X SE	0.1 mg/mL	Invitrogen (A20770)
Primary antibodies		
Anti-BRD4 (A-7), mouse monoclonal	2 µg/mL	Santa Cruz sc-518021
Anti-Human LEDGF, mouse	2 µg/mL	BD Biosciences (611714)
Anti-Histone H3K9me3, rabbit monoclonal	1 µg/mL	Abcam (ab176916)
Anti-Histone H3K27me3, rabbit monoclonal	5 µg/mL	Abcam (ab222481)
Anti-Histone H3K36me3, rabbit polyclonal	1 µg/mL	Abcam (ab9050)
Anti-Histone H3K36me2, rabbit monoclonal	9 µg/mL	Abcam (ab176921)
H3K9/14Ac antibody, rabbit polyclonal	0.4 µg/mL	Diagenode (C15410200)

Secondary antibodies		
Goat Anti-Mouse IgG, Alexa 488 dye	4 µg/mL	Invitrogen/Thermo (A11001)
Goat Anti-Rabbit IgG, polyclonal Atto647N dye	2 µg/mL	Sigma-Aldrich (40839)
**GELATION/ DIGESTION**		
Sodium Chloride	2 M	Fisher Chemical (S/3160/60)
Sodium Acrylate	8.625% (w/w)	Sigma-Aldrich (408220)
Acrylamide	2.5% (w/w)	Sigma-Aldrich (A9099)
N,N'-methylenebisacrylamide	0.15% (w/w)	Sigma-Aldrich (M7279)
Tetramethylenediamine (TEMED)	0.2%	Sigma-Aldrich (T7024)
Ammonium Persulfate (APS)	0.2%	Sigma-Aldrich (A3678)
Double-Edge breakable Razor	/	Electron Microscopy Sciences (#72004)
Tris	50 mM	Carl Roth (2449.1)
Ethylenediaminetetraacetic acid (EDTA)	1 mM	Sigma-Aldrich (E6758)
Guanidine HCl	0.8 M	Sigma-Aldrich (G3272)
Proteinase K	8 U/mL	New England Biolabs (P8107S)
Glass-bottom 6-well plate	/	Cellvis (P06-1.5H-N)

Supplementary Table 12. FIJI script to quantify LEDGF immunofluorescence intensity from raw images (pre- and post-expansion).

```
//Z-project with average intensity to have the full stack analyzed
//split channels and just save DAPI and LEDGF channel since we will do analysis on this channel


run("Z Project...", "projection=[Average Intensity]");
ImageName=getTitle();
run("Split Channels");//solitting channels
selectWindow("C1-"+ImageName);
rename("DAPI-"+ImageName);
selectWindow("C2-"+ImageName);
rename("LEDGF-"+ImageName);
selectWindow("C3-"+ImageName);
close()
selectWindow("DAPI-"+ImageName);


//Use the DAPI image to detect ROI and LEDGF image to measure intensity on
selectWindow("DAPI-"+ImageName);
run("Gaussian Blur...", "sigma=5");
run("Auto Threshold", "method=Default white");
run("Make Binary", "method=Default background=Default calculate black");
setOption("BlackBackground", true);
run("Convert to Mask", "method=Default background=Default calculate black");
```

```
run("Analyze Particles...", "size=70-Infinity show=Outlines display exclude add");

//check if selected ROI's are okay and delete the ones who are not

waitForUser("Do not save Results, if ready hit OK")



//open image where you want the intensity from

selectWindow("LEDGF-"+ImageName);

run("Show Overlay");

run("Select All");

roiManager("Measure");

waitForUser("Save Results, if ready hit OK")

run("Clear Results");

selectWindow("Results")

close()
```
